# Microscopic Analysis and Evaluation of Thermal Elevation and Wear of Drills for Implant Site Preparation: An In Vitro Study

**DOI:** 10.3390/ma17225524

**Published:** 2024-11-12

**Authors:** Lucia Memè, Fabrizio Bambini, Tommaso Pizzolante, Martina Principi, Francesco Sampalmieri, Stefano Mummolo

**Affiliations:** 1 Department of Specialist Clinical and Odontostomatological Sciences, Polytechnic University of Marche, 60121 Ancona, Italy; l.meme@staff.univpm.it (L.M.); f.sampalmieri@staff.univpm.it (F.S.); 2 Department of Life, Health and Environmental Sciences, University of L’Aquila, 67100 L’Aquila, Italy; tommaso.pizzolante@student.univaq.it (T.P.); martina.principi@student.univaq.it (M.P.); stefano.mummolo@univaq.it (S.M.)

**Keywords:** implant cutters, kit comparison, wear, temperature rise, usage time

## Abstract

Drilling for implant site preparation generates heat, which can cause bone necrosis if temperatures exceed 47 °C for over a minute. Factors influencing heat include drill size, speed, pressure, irrigation, and tool wear. Frequent drill replacement is essential, as wear from repeated use and sterilization affects performance. This study compared three pilot drills with similar designs from different manufacturers, testing each on pig ribs for 15 perforations after 15 sterilization cycles. Researchers measured temperature increase, drilling time, and surface wear. Results showed that drill no. 1 generated more heat than drills no. 2 and no. 3, though none reached critical temperatures. Drill no. 2 took the longest to reach the desired depth and displayed the most deformation. Findings highlight the importance of adhering to the recommended operational limits, suggesting that drills should be replaced after 15 cycles to ensure efficacy and patient safety.

## 1. Introduction

Implant surgical therapy involves multiple stages aimed at non-traumatically preparing the implant bed. Initially, the ridge is flattened using a rosette cutter with sterile irrigation. Following this, the cortical bone is penetrated with a small or lanceolate diameter rosette cutter. The surgeon then utilizes spiral cutters of increasing diameter to reach the implant site, employing an intermittent movement, while ensuring continuous irrigation [1,2]. The bone chips generated during this process can be recovered and reused. After each cutter’s use, the axis and depth of the implant site are verified using parallel pins, with any deviations corrected using subsequently larger diameter cutters. In cases of very dense bone, site tapping may be performed; although, this practice is less common due to the availability of self-tapping implants. Afterward, the depth and walls of the site are assessed, removing any bone fragments and irrigating with saline. The sterilized implant is then inserted into the prepared site at a low speed, applying a torque of 32 N-cm to evaluate the primary stability. Regarding this phase of implant insertion, Memè L. et al. [3] describe the structural changes of two different implant systems, external hexagon and internal hexagon, which were subjected to increasing torque values and, subsequently, observed and photographed to assess the potential consequences of these changes on the stability of the prosthesis over time. The preparation of the implant site is particularly critical in implant therapy, as it can significantly affect the healing and maintenance of the bone surrounding the implant [4]. The use of aggressive drills, known as countersinks, on the bone crest has been shown to cause resorption at the first thread when residual crests are smaller than 6 mm. The action of such drills can lead to excessive thinning of the vestibular, lingual, or palatal ridges, resulting in a reduction in the minimum thickness, as indicated in the scientific literature [5]. The positioning of the implant concerning the thickness of the soft tissues is also crucial to prevent bone resorption around the implant neck, necessitating careful consideration during implant insertion [6]. Notably, studies investigating the use of piezoelectric instruments in implant site preparation suggest that piezo surgery offers low levels of overheating of the bone surface and high precision during the initial instrument insertion. However, continuous irrigation and adherence to the manufacturer’s guidelines during instrument manipulation are essential to realizing these advantages [7]. Ultimately, the surgeon proceeds with the insertion of a screw “cap” or a “healing” screw, depending on the system and treatment stage. In transmucosal systems, soft tissues are sutured around the healing screw. These steps ensure the meticulous preparation of the implant site, which is vital for the success of the treatment [8,9].

Rotary cutters and tips are essential tools in implant therapy. Milling cutters are rotating instruments with a primary design (cylindrical, truncated conical, ball-shaped, etc.) [10] and a secondary design featuring tungsten carbide blades that vary in number and morphology [11,12]. The tips are abrasive tools that possess a working surface not geometrically defined. This surface is typically composed of diamond crystals arranged randomly and embedded in a nickel binder via an electrolyte bath. Each drill or cutter consists of several components, including the stem, which is inserted into the turbine or multiplier handpiece (red or blue ring), the working part responsible for dental material removal, and the head, the most apical portion of the instrument, which may or may not have a working function. Depending on the application, diamond cutters and bits come in various shapes and sizes. Tungsten carbide cutters (ISO 500) exhibit a hardness of 1600 HV (Vickers), compared to the 400 HV hardness of steel [13]. The secondary design of these cutters may feature helical, transverse, straight, or cross-cut blades. The number of blades varies [13,14,15]; an increased number of blades ensures a more uniform cutting action, while fewer blades enhance cutting capacity. As the number of blades increases, the ability to finish and polish also improves [16,17].

Cutters made of steel for surgical use are designed for mechanical applications and feature a stem with a contra-angle attachment, necessitating a suitable micromotor for operation. The correct insertion of these cutters into the handpieces is essential to avoid vibration, eccentric rotation, premature wear, and bending of the stem. It is advisable to use coolant to prevent bone necrosis in the absence of proper irrigation during use. The wear of these cutters significantly depends on the type and density of the bone being drilled, as harder bone results in faster tool consumption. It is recommended to replace these cutters after approximately 20 working cycles to ensure effective and safe cutting without compromising their performance [18].

In preparing the implant site, cutters are categorized based on their diameter, length, and configuration of turns. Preparation cutters may be cylinder conical, with a two-cutting helical geometry and diameters of up to 3.00 mm; larger diameters can accommodate an increased number of blades [19]. To facilitate identification during surgery, preparation tools can be equipped with colored rings, allowing surgeons to easily associate the cutter with the diameter of the chosen implant. The recommended number of rotations per minute (RPM) for drilling primarily depends on bone density. Typically, 800–1200 RPM is recommended for types I and II bone, while 500 RPM is advised for type III, and 800 RPM is recommended for type IV bone. Lanceolate implant cutters are designed to facilitate the preparation of the implant tunnel and are compatible with most implant systems, ensuring a quick and precise cut, while maintaining optimal balance and elasticity, even in the presence of bone D1. Pilot cutters are generally employed for types I and II bone, while their use is optional for types III and IV bone; the maximum speed of these cutters should be 800 RPM for types III and IV bone and 1200 RPM for types I and II bone [20,21]. This study aimed to compare the degree of wear and temperature rise of pilot drills belonging to kits from different implant companies [22,23].

This study provides valuable insights into the thermal dynamics and wear characteristics of dental drills used in implant site preparation. By evaluating the comparative wear and temperature elevation of different pilot drills under controlled conditions, the authors contribute essential data on how material properties, sterilization, and usage cycles impact drill performance and patient safety. This work serves to advance surgical tool design by emphasizing factors that mitigate thermal risk and wear-related degradation.

This study articulates specific research questions aimed at understanding the wear of pilot drills during dental implant site preparation and clarifies its distinct contribution to the field compared to previous work. The primary research questions addressed in this study include the impact of wear on pilot drills, specifically evaluating how wear affects temperature rise, usage time, and cutting accuracy, while considering the influence of multiple sterilization cycles. Additionally, the study investigates the temperature levels reached by different types of pilot drills during drilling, assessing how these temperatures vary among the tested models, with a critical goal of determining whether any of the drills exceed the safety threshold necessary to prevent bone necrosis.

The study’s distinct contribution lies in its direct comparison of different types of pilot drills from various manufacturers using a realistic biological model, specifically pig ribs. This approach enables a comprehensive assessment of drill wear and temperature rise through a detailed methodology, contrasting with prior research that has often focused on single drill models or systems. Furthermore, the examination of the effects of working and sterilization cycles leads to the recommendation of a maximum of 15 cycles to ensure clinical effectiveness, while minimizing overheating risks. The findings of this study enhance clinical practice by underscoring the importance of adhering to manufacturer guidelines to prevent damage to bone tissue.

In conclusion, this study not only evaluates the performance of various drills but also provides practical recommendations aimed at improving safety and tool longevity during implant surgery. This comparative and practical approach distinguishes the research by integrating safety parameters, such as critical temperatures, with aspects of clinical efficacy.

## 2. Materials and Methods

### 2.1. Study Design

The study employed three pilot drills with distinct specifications to assess their performance and wear characteristics. All cutters have a similar conformation: they are cylindrical cutters with two cutting edges.

Drill no. 1, constructed from stainless steel, has a diameter of 2.2 mm and is noted for its high resistance to wear and corrosion, with a maximum recommended speed of 800 rpm when adequate irrigation is provided. Drill no. 2 is made of carbide-coated stainless steel, features a 2.0 mm diameter, and operates optimally within a speed range of 900 to 1100 rpm. It is crucial to monitor the condition of its cutting edge frequently to prevent excessive heating. Drill no. 3, also 2.0 mm in diameter, is made from surgical-grade stainless steel with similar wear and corrosion resistance to drill no. 1 and operates at a maximum speed of 800 rpm. All drills were observed using a Zeiss Axio Zoom V16 microscope (Carl Zeiss Microscopy GmbH 07745, Jena, Germany) and a Neway+ 3D scanner was employed to obtain detailed digital models of their cutting surfaces before use.

For the biological model, pig ribs were selected due to their structural and density similarities to human bone, providing a realistic simulation of clinical drilling conditions. Each rib segment was securely fixed to a stand to ensure stability during drilling, facilitating a controlled assessment of drill performance across multiple perforation cycles.

In terms of equipment, an Eventek noncontact infrared thermometer, capable of measuring temperatures from −50 °C to 420 °C, was utilized to record drill temperatures during and after use. A surgical micromotor was set to a torque of 49 N-cm and was employed with a green ring handpiece to maintain consistent drill speeds and torque, ensuring uniform application of pressure throughout all trials.

The experimental methods involved a drilling protocol where each drill performed 15 perforations at a depth of 15 mm on different segments of the pig ribs. Initially, a lanceolate starter drill was used to create a guide hole at 1000 rpm. Following this, each pilot drill was tested at a maximum speed of 800 rpm with continuous irrigation provided at a flow rate of 50% physiological saline. To maintain consistency, all drilling procedures were conducted by the same operator, and drill temperatures were recorded at regular intervals.

Temperature and usage time measurements were taken immediately after each drilling cycle at both the tip and at a depth of 15 mm using the Eventek infrared thermometer (Shenzhenshi Huitianyi Electronic Co., Ltd., Shenzhen City, China). A total of thirty temperature measurements per drill were conducted, ensuring accuracy by directing infrared rays at specific distances and angles. Additionally, a second operator measured the time required for each drill to reach the target depth over the 15 perforations using a stopwatch (Sweden & Martina, Via Veneto, 10, 35020 Due Carrare PD, Italy).

Post-use, all drills underwent 15 sterilization cycles and were subsequently re-examined under the microscope. A second round of 3D scanning was performed with the Neway+ scanner, followed by surface analysis using Zeiss Inspect software (Zeiss Quality Suite in v5.3) to compare pre- and post-use STL files. This analysis focused on the working length of each drill (15 mm), assessing both maximum and minimum surface discrepancies, while identifying wear patterns. Surface discrepancies were measured with a threshold distance of 0.5 mm, and color-coded mapping was utilized to indicate regions of wear.

The sterilization procedure involved subjecting each drill to 15 cycles of autoclave sterilization, following the standard clinical sterilization protocol, which included specified temperature and duration settings according to the autoclave specifications. This procedure was essential for evaluating drill wear under repeated sterilization conditions, helping to assess the resilience of each material and the consistency of cutting performance across multiple uses.

Drill no. 1 is made of stainless steel with high resistance to wear and corrosion, is autoclavable, and has a diameter of 2.2 mm.

**Figure 1:**
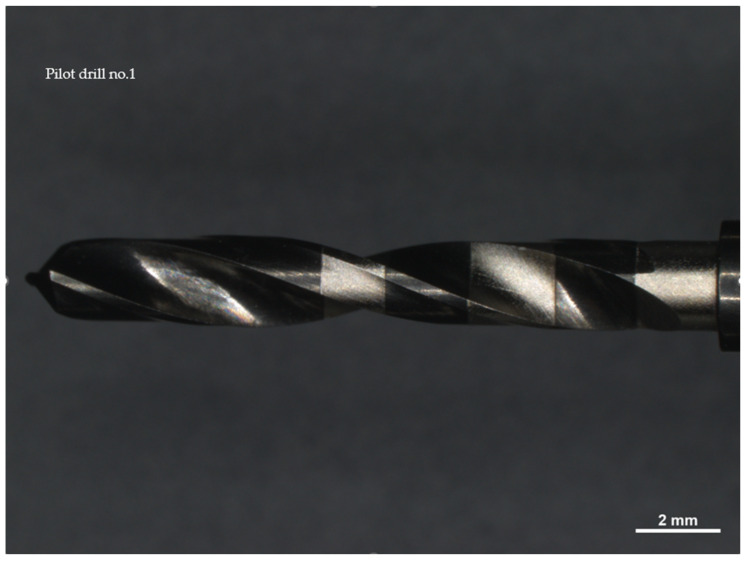
Drill no. 1 is an autoclavable stainless steel drill with a diameter of 2.2 mm. It operates at a maximum speed of 800 rpm with irrigation.

The drill must be used at a maximum speed of 800 rpm, with adequate irrigation, up to the depth mark corresponding to the selected implant length (Figure 1).

This cylindrical cutter is made of carbide-coated stainless steel and has a diameter of 2 mm.

**Figure 2:**
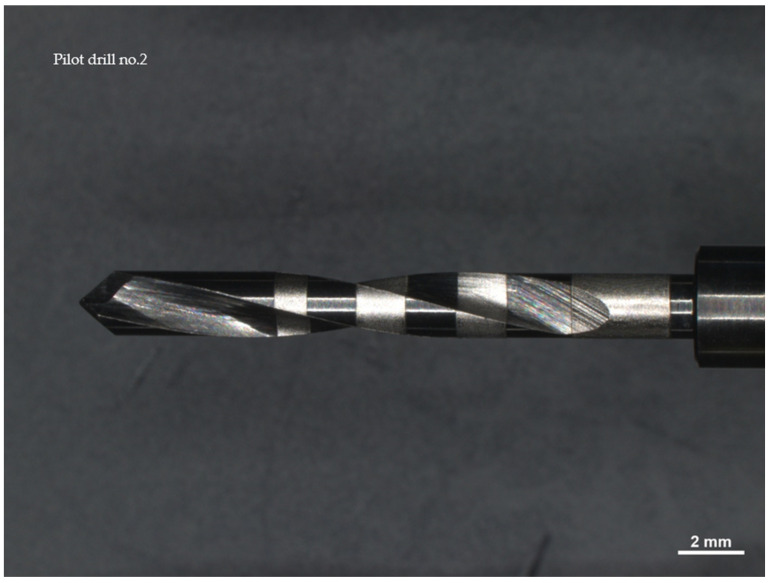
Drill no. 2 is a cylindrical cutter with a diameter of 2 mm, made of carbide-coated stainless steel. Recommended cutting speed: 900–1100 rpm.

The recommended cutting speed is in the range of 900–1100 rpm; however, it is necessary to check the wear of the cutting edge to avoid overheating and/or tearing fabrics (Figure 2).

**Figure 3:**
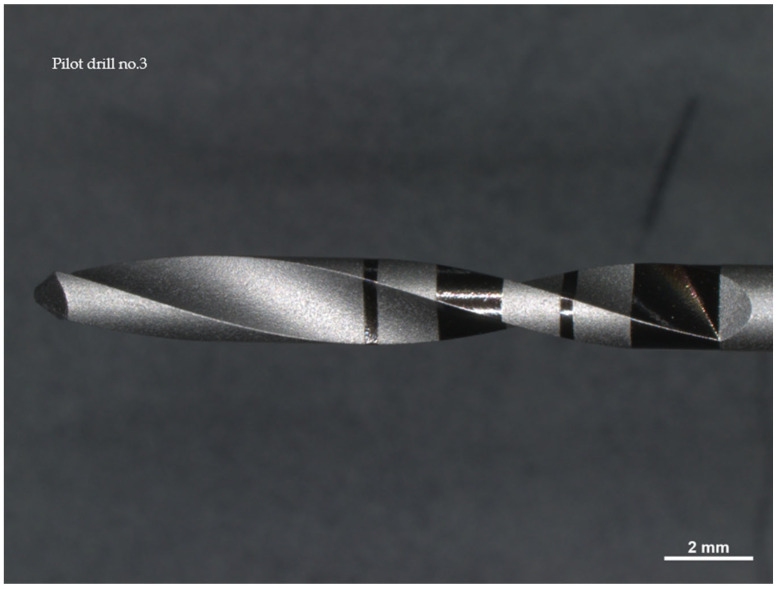
Drill no. 3 is constructed from stainless surgical steel and has a diameter of 2 mm.

Drill no. 3 is made of stainless surgical steel characterized by high resistance to corrosion and wear; it has a diameter of 2 mm (Figure 3).

**Figure 4:**
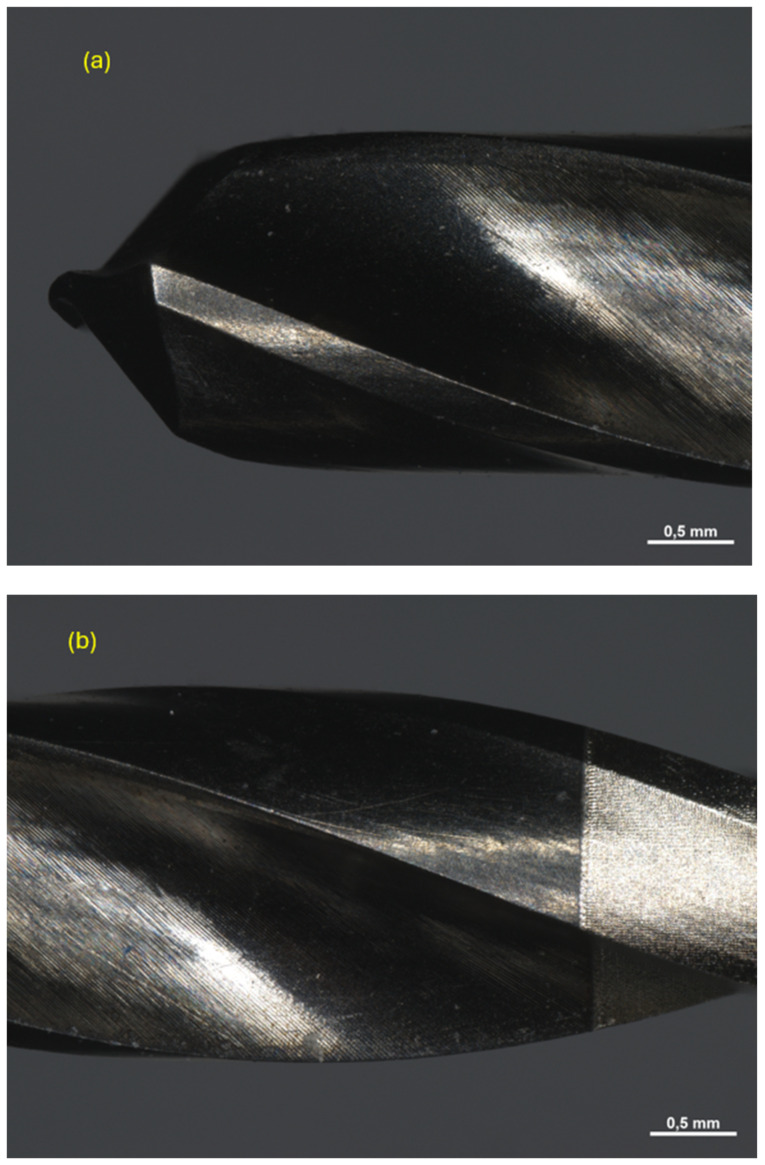

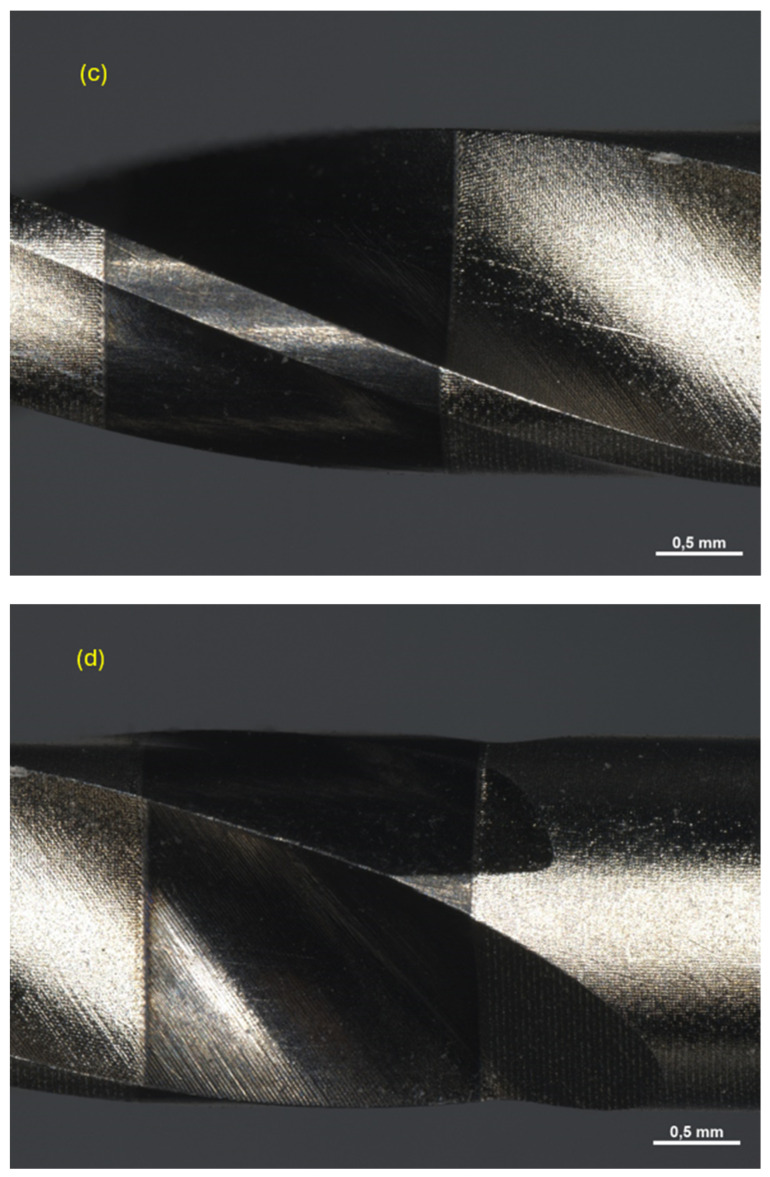
Photos of pilot drill no. 1 obtained under the microscope before use. (**a**) Image of the drill at tip level, (**b**,**c**) images of the drill in the middle, (**d**) image of the drill at the end part.

**Figure 5:**
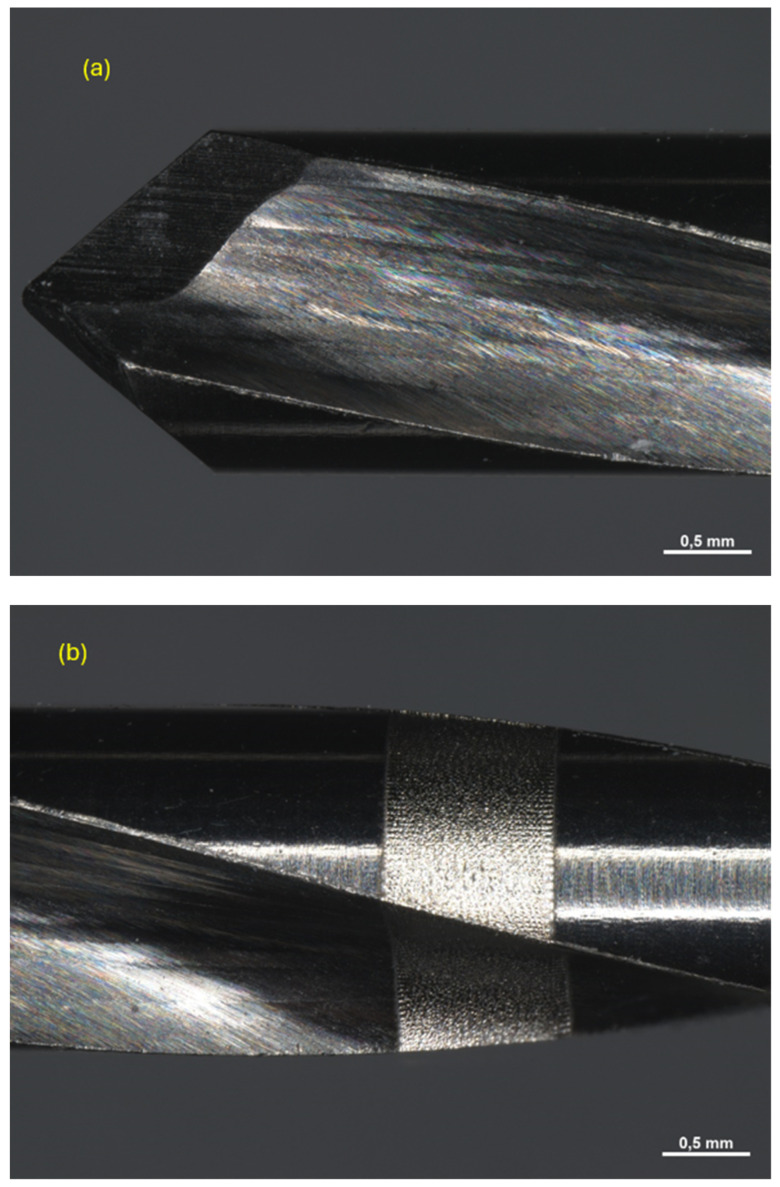

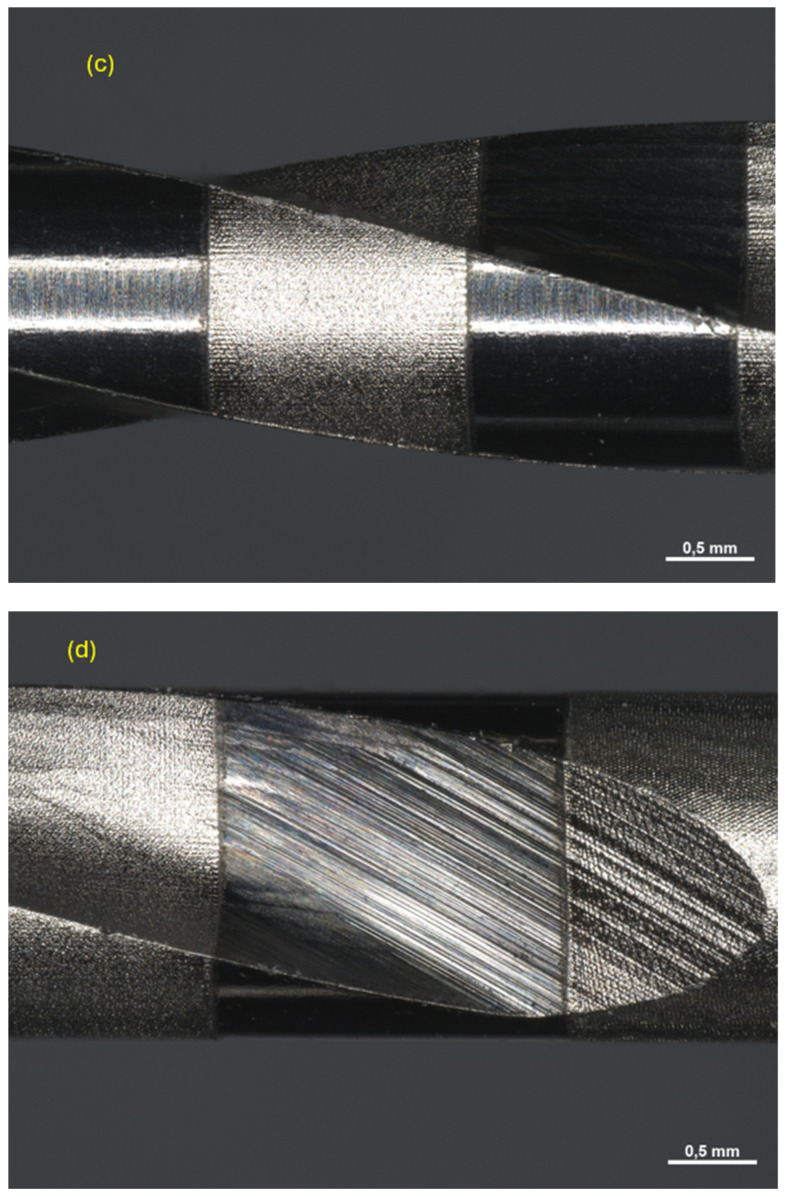
Photos of pilot drill no. 2 obtained under the microscope before use. (**a**) Image of the drill at tip level, (**b**,**c**) images of the drill in the middle, (**d**) image of the drill at the end part.

**Figure 6:**
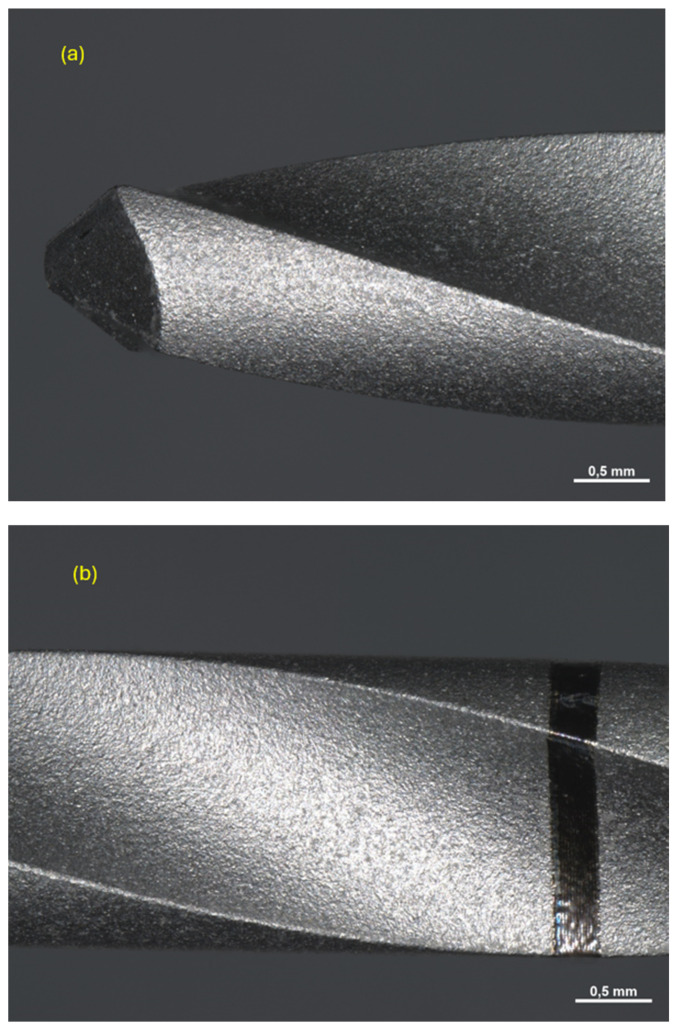

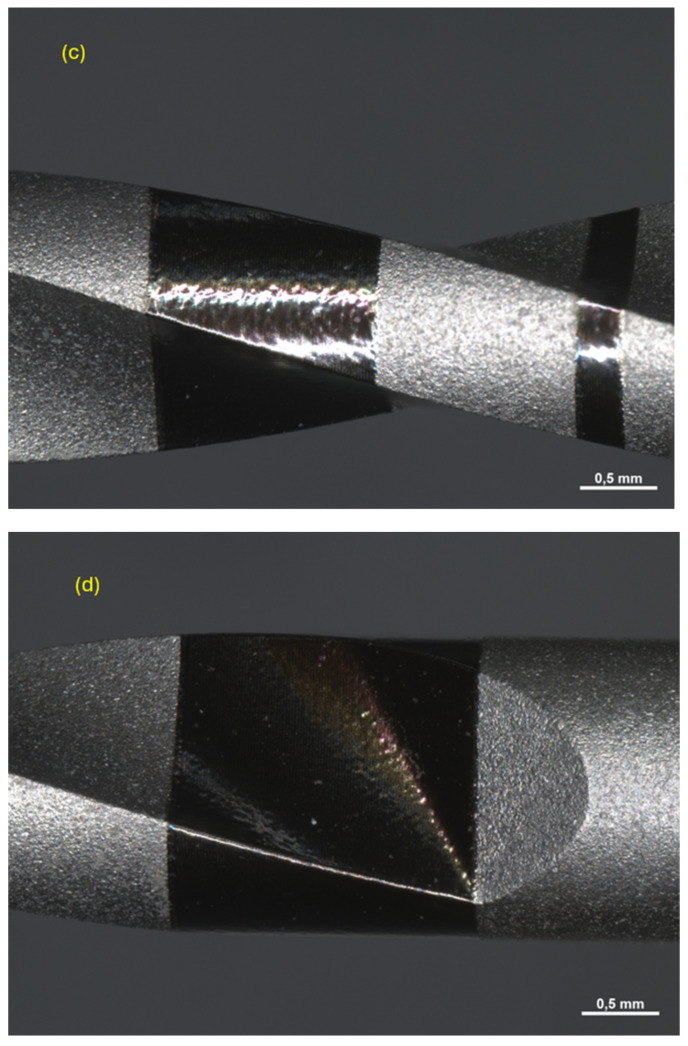

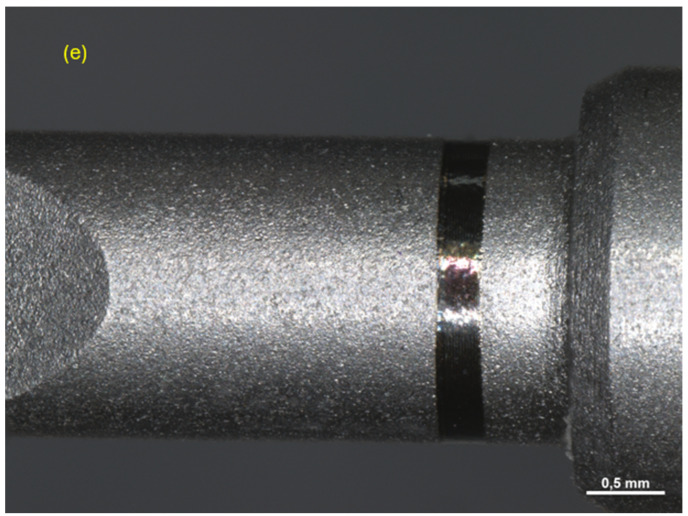
Photos of pilot drill no. 3 obtained under the microscope before use. Photos of pilot drill no. 2 obtained under the microscope before use. (**a**) Image of the drill at tip level, (**b**–**d**) images of the drill in the middle, (**e**) image of the drill at the end part.

All drills were observed under a Zeiss Axio Zoom V16 microscope (Carl Zeiss Microscopy GmbH 07745, Jena, Germany, single-path zoom microscope for stereo, reflected light, transmitted light (bright-field), and fluorescence acquisitions with optical sectioning) before use (Figure 4, Figure 5 and Figure 6).

**Figure 7:**
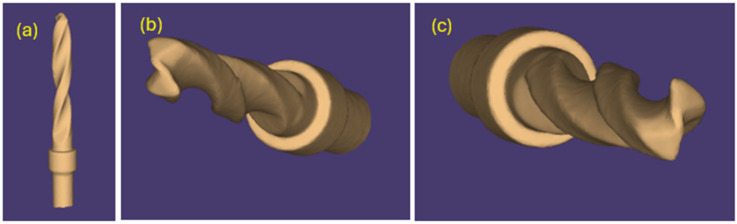
(**a**–**c**) Images obtained from the STL file of pilot drill no. 1 pre-use.

**Figure 8:**
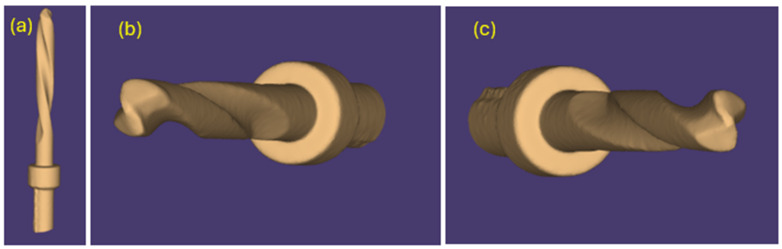
(**a**–**c**) Images obtained from the STL file of pilot drill no. 2 pre-use.

**Figure 9:**
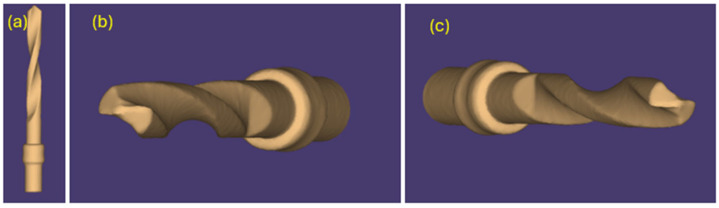
(**a**–**c**) Images obtained from the STL file of pilot drill no. 3 pre-use.

Pilot drill no. 1:

Pilot drill no. 2:

Pilot drill no. 3:

The same pilot cutters were also pre-scanned using a 3D scanner Neway+ Open Technology (Headquarters Via Giovanni XXIII, 75A 25086 Rezzato, BS, Italy). Prior to scanning, the surface of the instruments was treated with Renfert-Scanspray classic, a fine-grained 3D matting spray for extraoral scanning of shiny or highly reflective surfaces, with the aim of constructing a suitable mesh; the use of this spray might have slightly altered the scanner’s reading of the surface. From the scanning of the cutters, STL files were generated and visualized via the Exocad WebView platform in v1.6.18 (Figure 7, Figure 8 and Figure 9).

A standardized cooling period was enforced before each scanning session, allowing all drills to return to ambient temperature and minimizing any temperature-related discrepancies due to thermal expansion. Pre- and post-use temperatures were verified with an infrared thermometer immediately prior to scanning, confirming that each drill was at room temperature. This consistency ensured that thermal expansion effects were negligible at the time of measurement, preventing variations that could otherwise distort wear assessment.

The scanning procedures were conducted in a controlled laboratory environment with stable ambient temperature and humidity, eliminating temperature fluctuations that might affect the dimensions of the drills. At room temperature, metals, such as stainless steel and carbide—common materials for dental drills—exhibit negligible thermal expansion, ensuring that the drills maintained stable dimensions for accurate comparison.

Additionally, the scanning equipment was calibrated to standard operating conditions, accounting for any minor residual temperature differences and controlling for potential thermal artifacts. Infrared thermometry provided direct confirmation that drill temperatures were consistent pre- and post-use, avoiding expansion-related variance and enabling a precise and reliable wear analysis.

Through this careful temperature control and verification, the study effectively prevented thermal expansion from influencing the results, leading to a more accurate and consistent assessment of drill wear.

To enhance the understanding of the methodology utilized in this study, a more comprehensive explanation of the 3D scanning approach, along with its purpose and rationale, is essential.

The 3D scanning approach is fundamental to accurately assessing wear and deformation on the pilot drills both before and after repeated use. The authors implemented a high-resolution 3D scanning protocol to capture the detailed surface topography of each drill, allowing for the precise quantification of wear. The primary goal of employing 3D scanning in this research was to provide an objective and quantitative assessment of wear on the drill tips and cutting edges. Traditional wear measurements, such as visual inspections, are often hindered by subjective interpretation and the absence of quantitative depth. In this study, 3D scans facilitated the generation of precise digital models of each drill surface, enabling a detailed analysis of any changes in material thickness and surface structure following use.

Prior to each drill’s utilization, a baseline 3D scan was conducted to establish a digital model of its initial condition. This baseline serves as a reference for detecting and measuring any material loss or surface alterations that occur after repeated drilling cycles. To enhance scanning accuracy, each drill was treated with a 3D matting spray that minimized reflections from metallic surfaces, which could distort measurements. After a series of 15 perforations and subsequent sterilization cycles, each drill was rescanned under identical conditions. This post-use scan provides a comparative dataset for identifying alterations in surface structure, thickness, and geometry.

The pre- and post-use 3D models of each drill were digitally overlaid using Zeiss Inspect software, a process known as “surface comparison.” This technique allows the software to map any deviations between the two scans, with areas of material loss appearing as depressions or thinning, while material buildup or deformation is indicated by increased thickness. The software calculates maximum and minimum discrepancies on the drill surfaces, reporting these as distance measurements in millimeters. This method offers a highly accurate assessment of wear, capable of detecting changes as small as 0.01 mm, far exceeding the resolution of visual inspection.

The rationale for the 3D scanning approach includes several key aspects. First, precision is crucial for identifying microscopic wear patterns that can significantly affect drill performance and safety in surgical applications; even small changes in cutting edge thickness or tip geometry can lead to inefficiencies, overheating, and compromised cutting action. Second, reproducibility is enhanced by creating a digital record of each drill’s surface before and after use, which allows for consistent analysis across multiple tools and use cycles, thereby making results more reliable than manual or observational assessments. Finally, objective comparison is achieved through the surface mapping of digital scans, which standardizes the wear assessment and minimizes subjective bias. This objective quantification is essential when comparing different drill materials or designs, establishing a uniform basis for comparison.

In conclusion, the integration of 3D scanning in this study enables a precise, objective, and reproducible assessment of wear on pilot drills, marking it as a crucial methodological component. By employing this approach, the authors effectively quantify minute surface changes, directly correlating wear patterns with performance outcomes, such as drilling time, temperature rise, and cutting efficiency. This methodology enhances the study’s reliability and relevance in clinical contexts, where maintaining tool integrity is vital for successful patient outcomes.

Pilot drill no. 1:

Pilot drill no. 2:

Pilot drill no. 3:

### 2.2. Surgical Procedure

The temperature of the pre-use cutters was measured using an Eventek device (noncontact infrared thermometer, temperature range of −50–420 °C) by directing the infrared rays directly onto the instruments from a standard distance [24,25].

**Figure 10:**
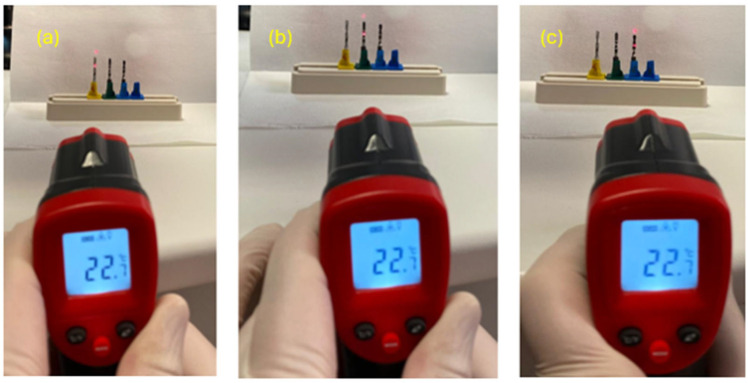
The measurements taken on pilot drills no. 3 (**a**), no. 2 (**b**), and no. 1 (**c**).

These measurements showed that the temperature before use was the same for all cutters (22.7 °C) (Figure 10).

**Figure 11:**
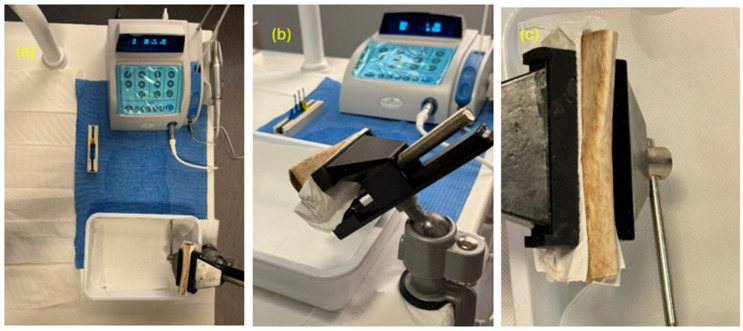
(**a**–**c**) Set of the study.

The drills were used, in the same number of passages, on pig ribs fixed on a stand, using a company-independent surgical micromotor and a green ring handpiece (Sweden & Martina, Via Veneto, 10, 35020 Due Carrare PD). The torque was set to 49 N-cm, and all instruments were combined during use with irrigation using a 50% physiological saline flow (Figure 11) [26,27,28].

**Figure 12:**
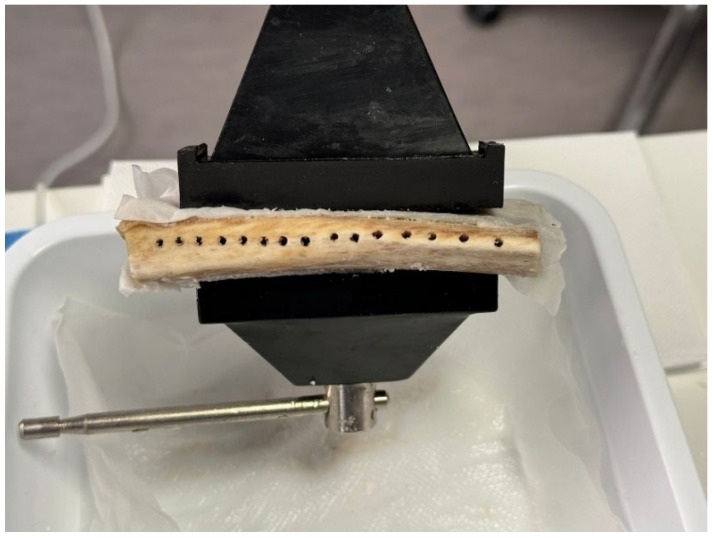
The initial cutter processed all samples at 1000 rpm, while pilot cutters were tested at 800 rpm for 15 passes with irrigation to a depth of 15 mm.

The initial lanceolate cutter, which was not the subject of this study, was used on all samples at a speed of 1000 rpm, while the pilot cutters were tested after 15 passes each at a maximum speed of 800 rpm, with external irrigation, to a depth of 15 mm (Figure 12). The entire milling procedure was performed by the same operator after test drilling.

**Figure 13:**
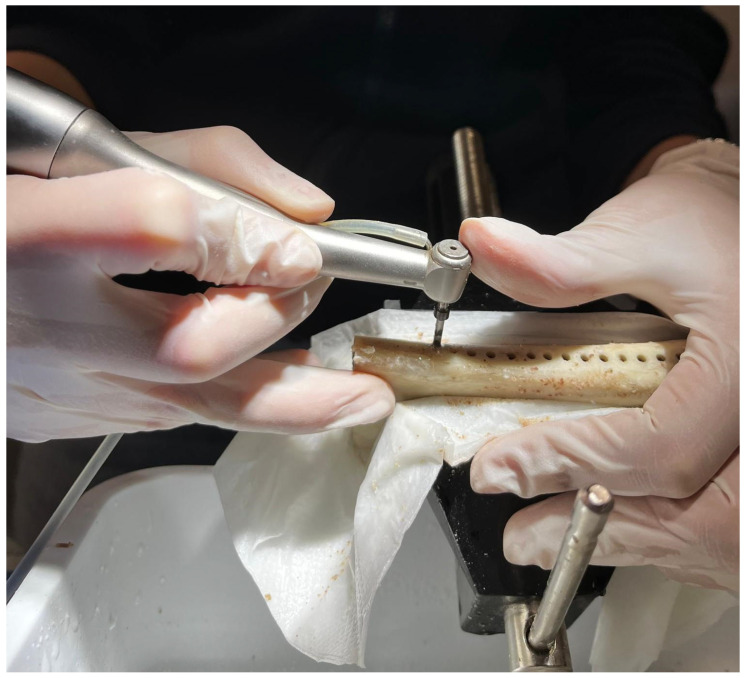
A lance cutter drilled 15 holes on each rib, spaced ≥3 mm apart, totaling 45 holes across three ribs. Each sample used a different pilot drill.

On each rib, 15 holes were drilled with a lance cutter at a distance of ≥3 mm, making a total of 45 holes that were distributed over three ribs; therefore, a different pilot drill was used on each sample (Figure 13).

### 2.3. Data Collection and Evaluation

**Figure 14:**
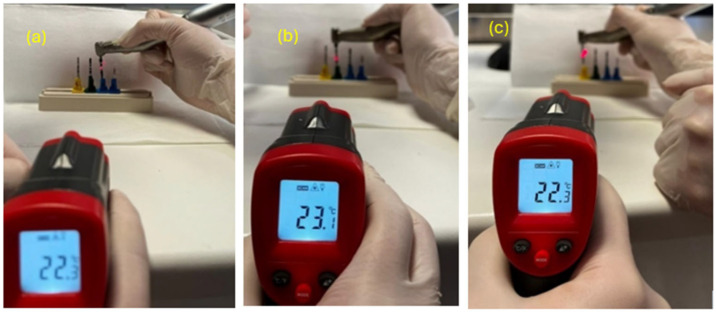
The measurements taken on pilot drills no. 1 (**a**), no. 2 (**b**), and no. 3 (**c**).

Immediately after the use of each individual cutter, their temperature was measured by a second operator using the Eventek equipment by directing the infrared rays at the tip of the instrument and at 15 mm from a standard distance; a total of 30 measurements per pilot cutter were then taken (Figure 14).

Below are some examples of how the measurements were carried out.

A third operator also measured the usage time of these drills with a stopwatch from the start of drilling until the working length was reached, for a total of 15 measurements per drill.

Models of contact interaction and destruction mechanisms in biomaterial drilling encompass several key processes that elucidate how the properties of biomaterials and drilling conditions contribute to drill degradation. The adhesive wear model illustrates the bonding that occurs at the contact surface between the biomaterial and the drill. As the drill rotates, microscopic areas of adhesion form and subsequently break, leading to the transfer of surface material from the biomaterial to the drill. This bonding mechanism increases friction and contributes to thermal elevation in the contact zone, thereby accelerating wear. In addition, the abrasion and microcutting model describes how hard particles or crystallites within the biomaterial act as cutting tools, gouging or scraping the drill surface. This interaction generates grooves and microscratches on the drill, gradually reducing its sharpness. Abrasion is particularly significant in harder biomaterials, resulting in pronounced tool degradation. The thermal fatigue and microcracking model focuses on the effects of thermal cycling during drilling, where fluctuations in temperature induce repeated expansion and contraction in the drill material. These cyclic stresses lead to microcracking, which propagates through the tool’s surface over time. This mechanism is exacerbated in biomaterials with low thermal conductivity, where heat accumulates rapidly at the drill–biomaterial interface.

**Figure 15:**
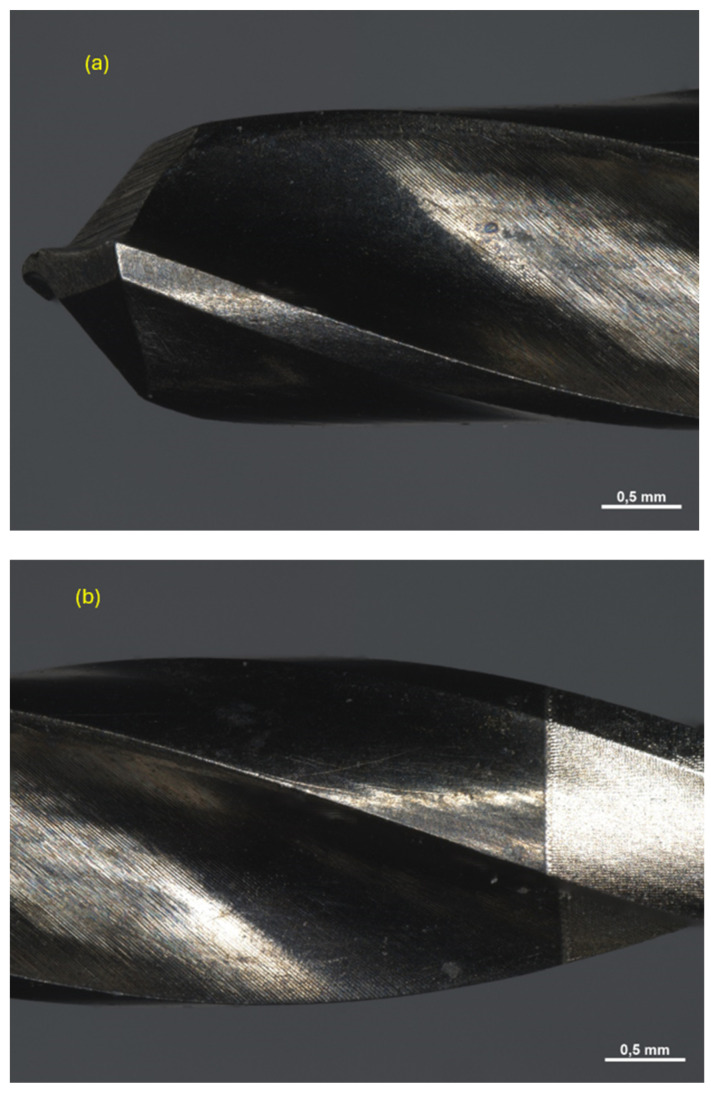

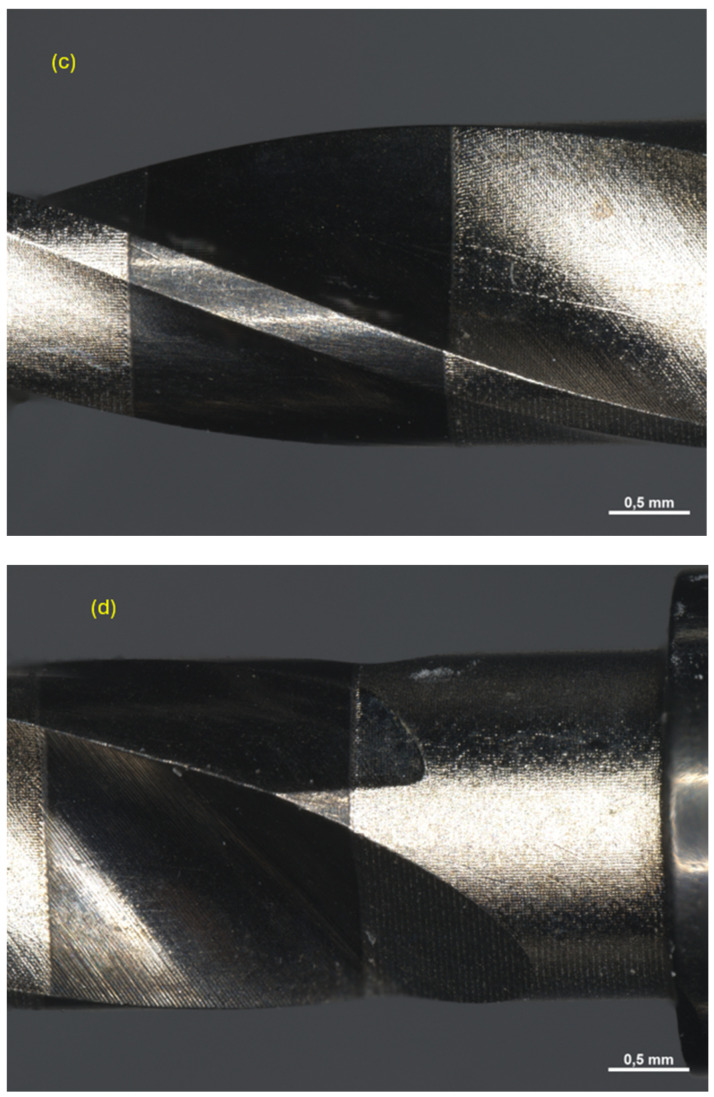
Photos obtained under the microscope of pilot drill no. 1 (**a**) after use and sterilization cycles at tip level (**b**), in the middle (**c**), and at the end part (**d**).

**Figure 16:**
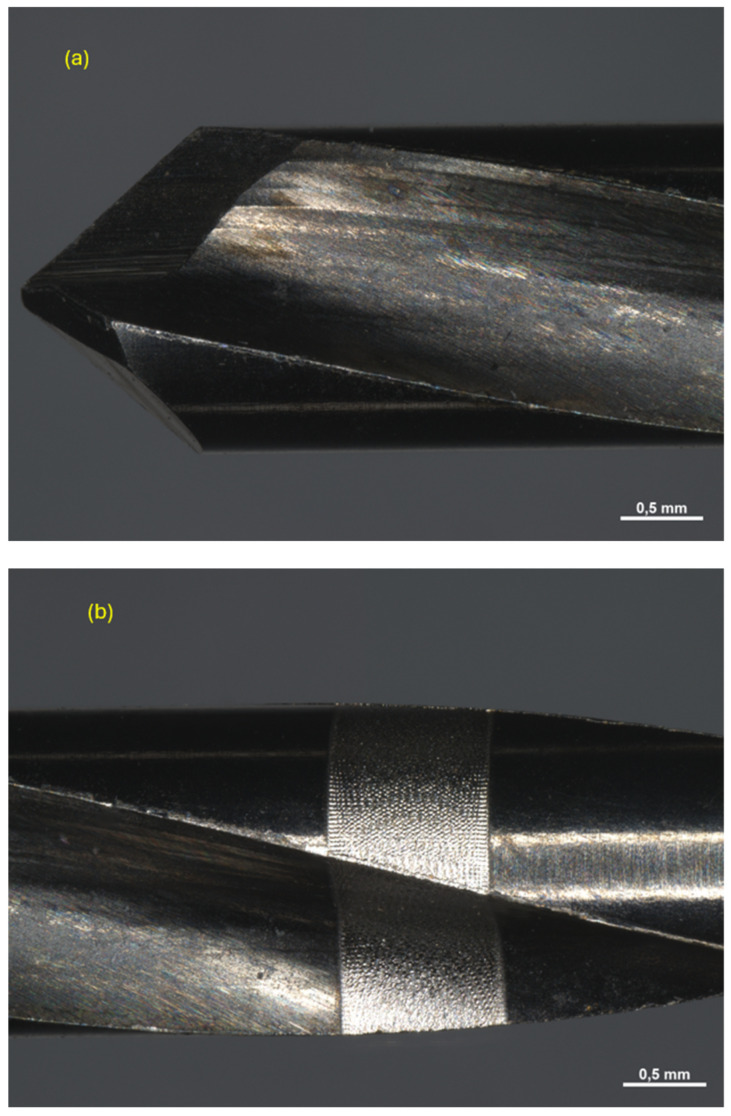

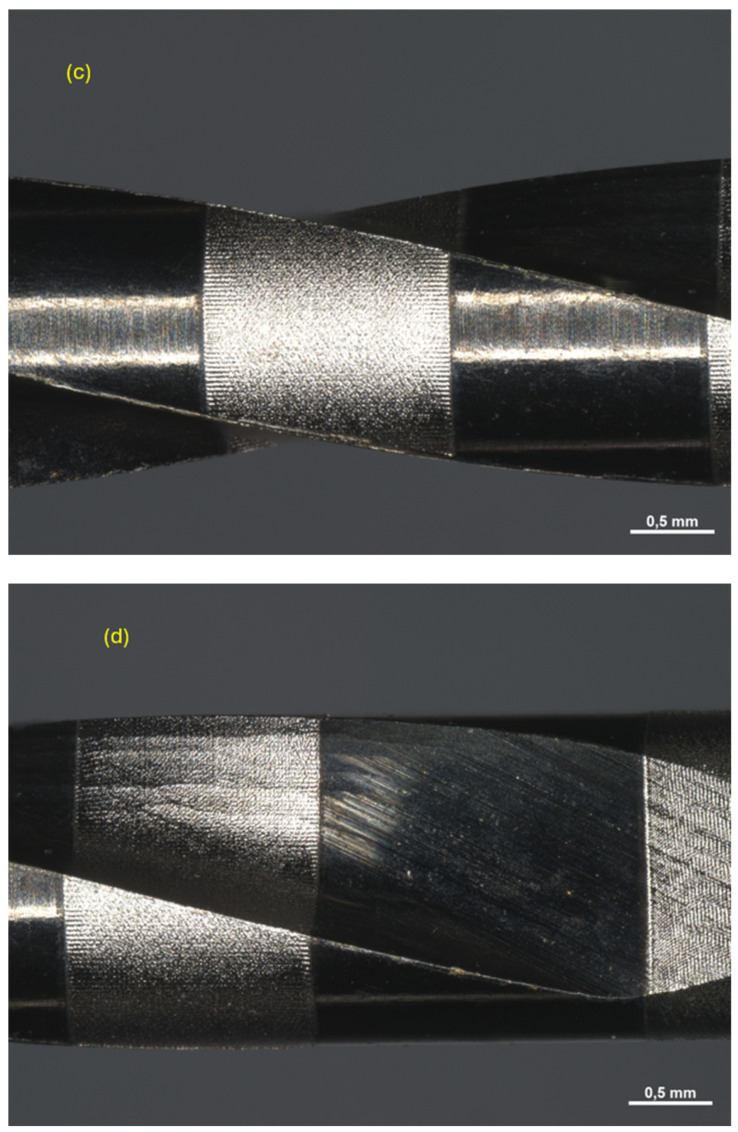

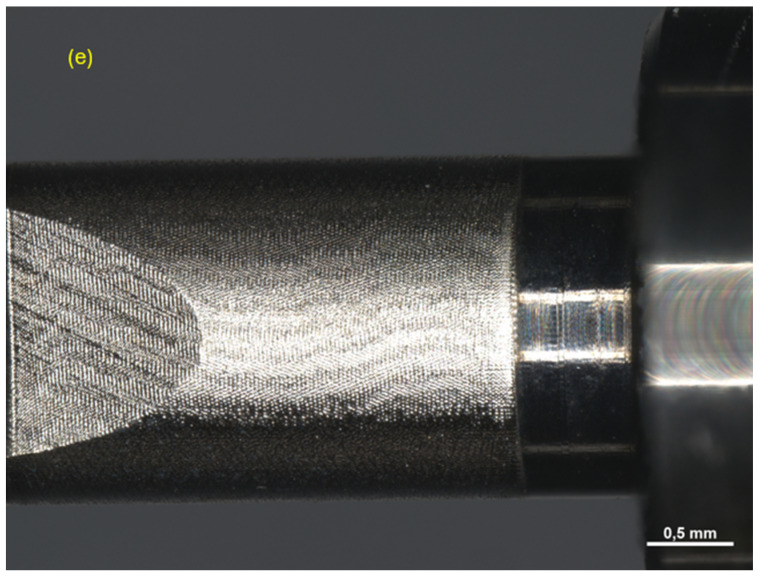
Photos obtained under the microscope of pilot drill no. 2 (**a**) after use and sterilization cycles at tip level (**b**), in the middle (**c**,**d**), and at the end part (**e**).

**Figure 17:**
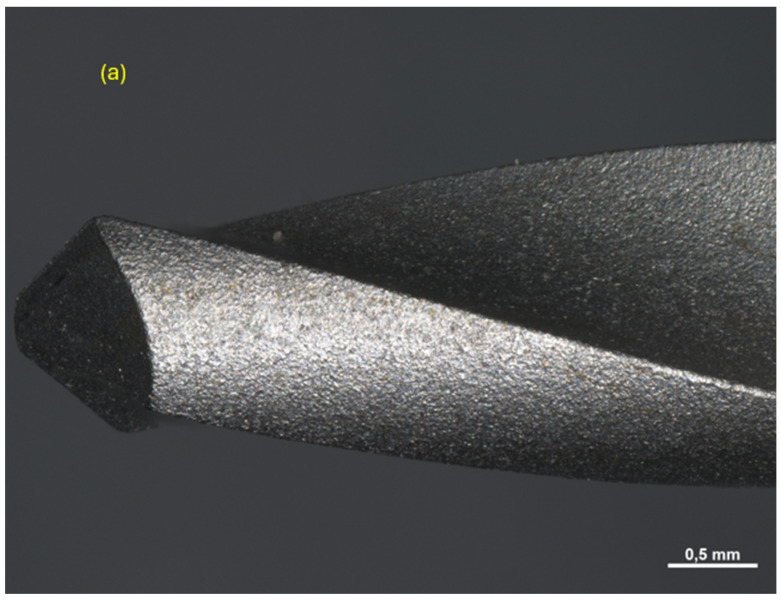

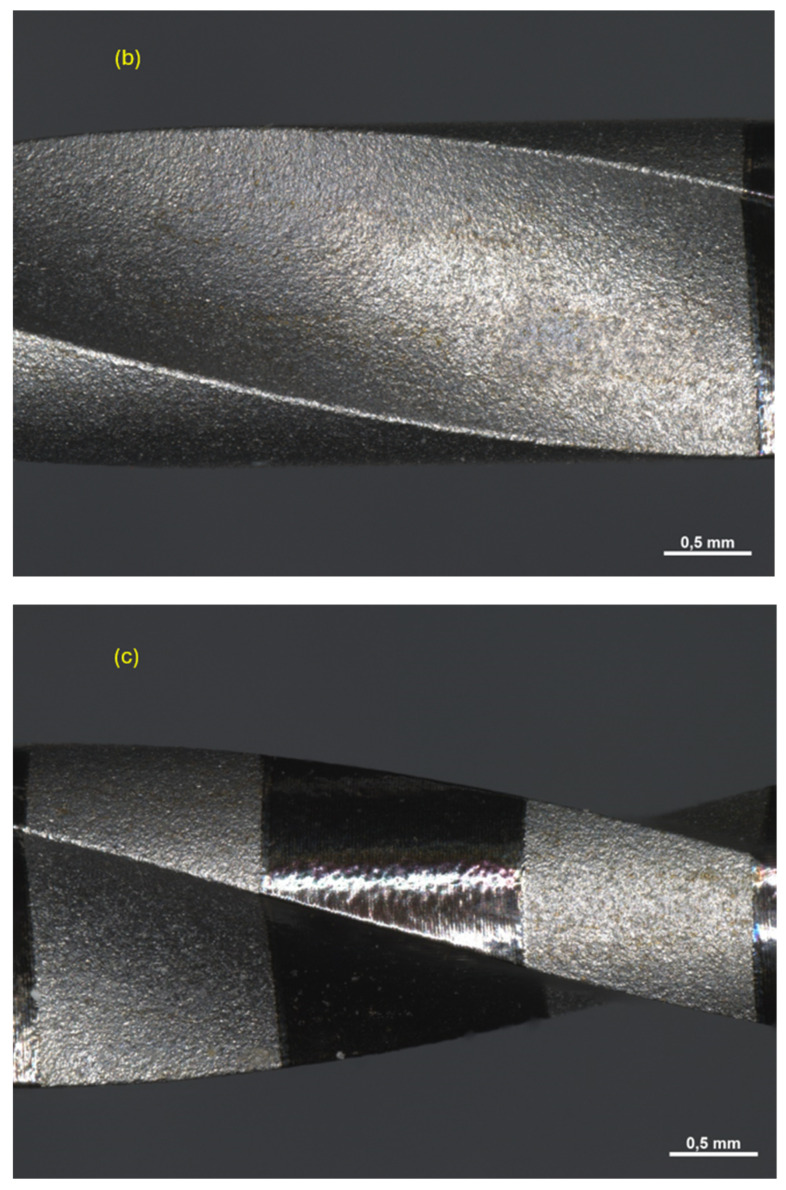

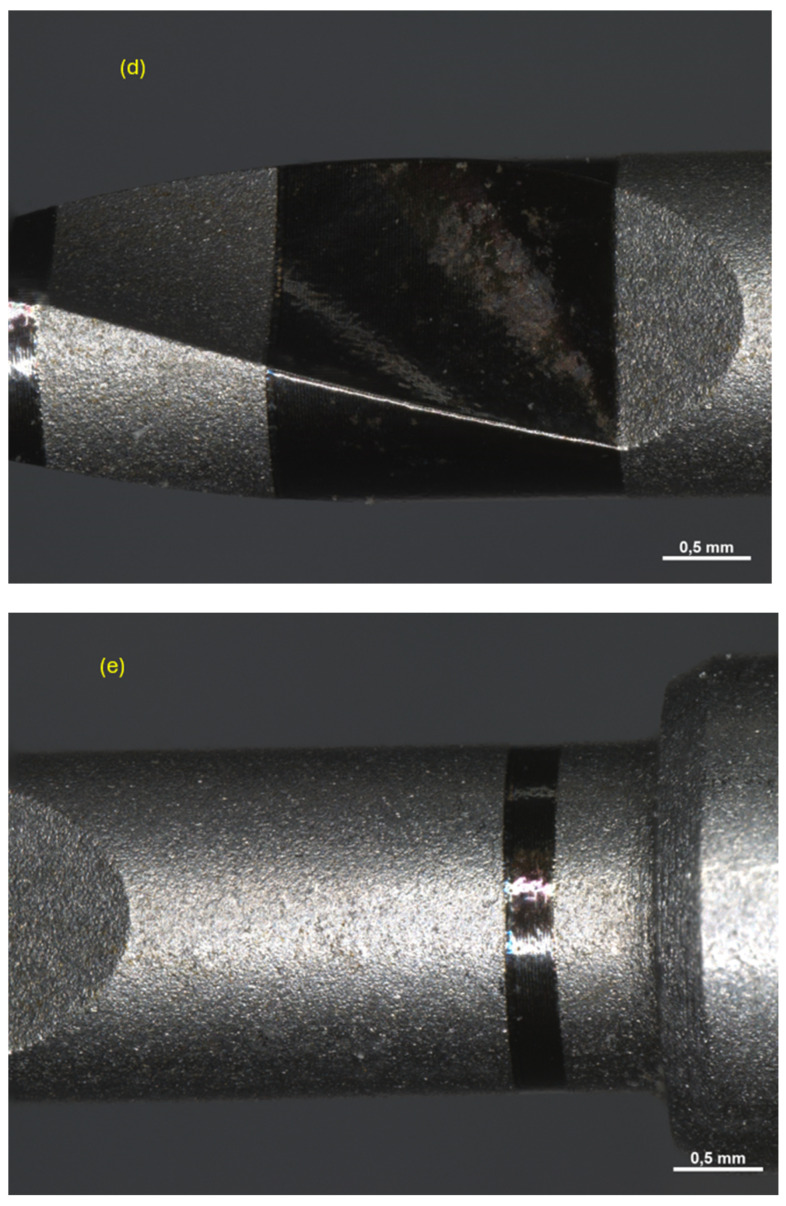
Photos obtained under the microscope of pilot drill no. 3 (**a**) after use and sterilization cycles at tip level (**b**), in the middle (**c**,**d**), and at the end part (**e**).

After use, all instruments were finally subjected to 15 sterilization cycles each and observed again under the microscope (Figure 15, Figure 16 and Figure 17). A detailed study of the phenomena occurring at the micro level within the drilling area enables the identification of the specific contributions of each material component to the overall wear process experienced by the drill tip. By isolating the effects of individual components, it becomes possible to understand how factors, such as hardness, thermal conductivity, and microstructural composition, influence wear patterns. This granular insight is critical for optimizing drill materials and designs that can better withstand repetitive mechanical stresses, thus extending tool life and improving surgical outcomes.

To elucidate the interaction mechanics between the biomaterial and the drill, a schematic model can be established that highlights key phases in the contact process. This model considers both mechanical and thermal interactions at the biomaterial–drill interface and comprises several critical elements. The first element is the dynamics of the contact zone, where the cutting edges of the drill engage with the biomaterial surface. This primary interface experiences immediate mechanical and thermal stresses due to high-pressure contact and rapid relative motion, which initiates frictional heat generation and material removal. The second element involves the formation of a thermal gradient. Due to the thermal properties of the biomaterial, a gradient develops with elevated temperatures concentrated near the drill tip. This thermal gradient affects heat dissipation and influences the wear rate, typically resulting in more intense wear in the areas subjected to sustained high temperatures. The third component of the model is the generation and transfer of frictional heat. As the drill penetrates the biomaterial, friction produces localized heating, and the schematic illustrates the transfer of this heat into both the biomaterial and the drill. This aspect underscores the importance of effective heat management to prevent thermal damage to the surrounding tissues. Additionally, the model identifies the distribution of thermal stress, highlighting the zones of thermal expansion and contraction that occur as heat cycles between the drill and the biomaterial during the cutting process. These thermal fluctuations contribute to stress accumulation, which can lead to fatigue and potential microcracking in both the drill and the material. The final component of the schematic enables the simulation of heat distribution on the contact surface, mapping the product–drilling surface interface. This visualization is essential for predicting and controlling temperature distribution, which is crucial for minimizing heat-induced degradation in both the drill and the biomaterial. Overall, this model integrates the mechanical and thermal aspects of the interaction process, providing valuable insights into the dynamics of biomaterial drilling (Table 1).

**Figure 18:**
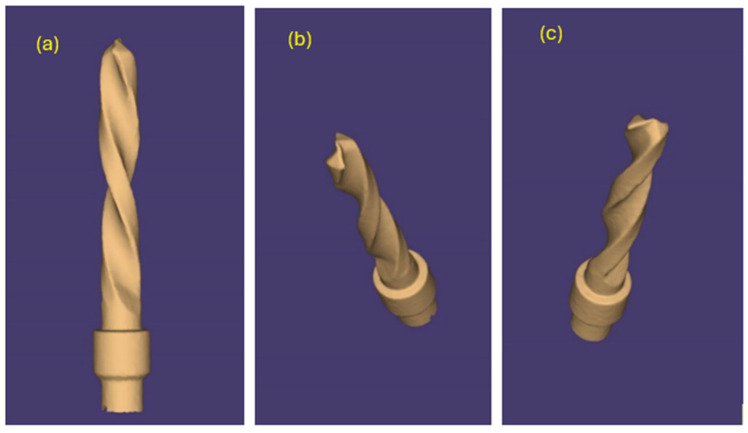
(**a**–**c**) Images obtained from the STL file of pilot drill no. 1 after use and sterilization cycles.

**Figure 19:**
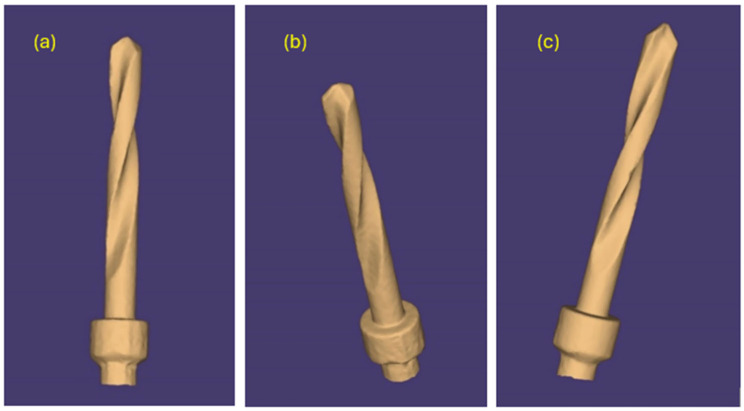
(**a**–**c**) Images obtained from the STL file of pilot drill no. 2 after use and sterilization cycles.

**Figure 20:**
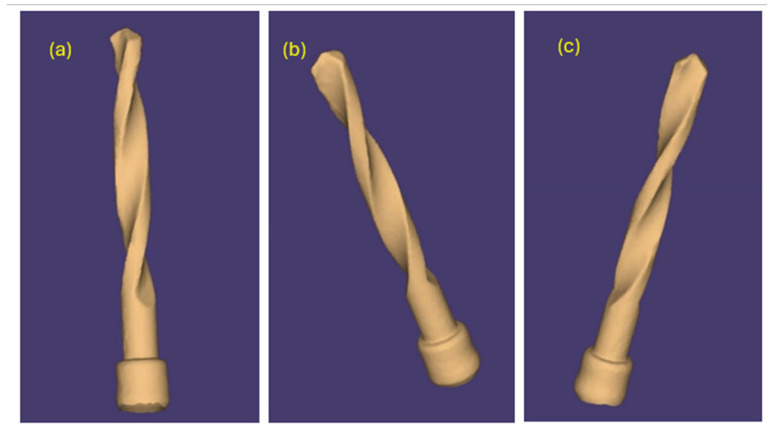
(**a**–**c**) Images obtained from the STL file of pilot drill no. 3 after use and sterilization cycles.

Pilot drill no. 1:

Pilot drill no. 2:

Pilot drill no. 3:

The pilot cutters were scanned again after use with a Neway+ Open Technology 3D scanner (Operational Headquarters Via Giovanni XXIII, 75A 25086 Rezzato (BS)). Prior to scanning, the surface of the instruments was treated with Renfert-Scanspray classic. From the scanning of the cutters, STL files were generated and visualized via the Exo WebView platform (Figure 18, Figure 19 and Figure 20).

Pilot drill no. 1:

Pilot drill no. 2:

Pilot drill no. 3:

Subsequently, using the Zeiss Inspect optical 3D software in version 5.3 (Carl Zeiss Industrielle Messtechnik GmbH, Carl-Zeiss-Str. 4-54 73447 Oberkochen, Germany), three-dimensional scans of the pilot cutters before and after use were compared, and the STL files were overlaid to assess the degree of wear three-dimensionally (22). The area selected for analysis corresponded to the area where the cutter was used (working length of 15 mm). Specifically, a comparison was conducted between the STL files of the pilot cutters pre- (imported by selecting the CAD body mode) and post-use (imported by selecting the Mesh mode); pre-alignment was then implemented for a surface comparison on CAD at a maximum distance of 0.5 mm; finally, the surface corresponding to the working length (15 mm) was selected for each pilot cutter, and the results shown in the images below were obtained.

The scanning resolution employed in this study is adequate for measuring drill wear based on several key considerations. The 3D scanning technology utilized offers a resolution of up to 0.01 mm (10 μm), which is precise enough to detect even minimal surface changes in the drill, critical for assessing tip sharpness and blade integrity. Given that only 15 perforations were performed per drill, the resulting wear remains moderate; however, the sensitivity of the scanning resolution allows for the capture of subtle surface alterations resulting from moderate use, such as minor thickness variations and deformations that may not be visible to the naked eye. Additionally, this resolution facilitates the detection of non-uniform wear across the drill’s working surface, particularly at edges or tips where pressure and friction are highest. This capability enables an assessment of how wear develops progressively in critical areas, offering insights into material durability and the effectiveness of drill design. The accuracy of the scans allows for precise alignment and direct comparison between pre- and post-use drill surfaces through digital overlay techniques, significantly reducing error margins and enabling reliable measurements of discrepancies that may arise after a moderate number of perforations. In clinical settings, even minimal wear can have implications for drill performance and patient safety. Therefore, the ability to detect wear at the micrometer scale ensures that drills adhere to clinical standards, especially in contexts where overheating or decreased cutting efficiency could negatively affect osseointegration. Furthermore, the data obtained at this resolution can support recommendations for drill replacement after a specified number of uses. By identifying wear at fine scales, this methodology establishes a foundation for defining replacement cycles that maintain optimal operational efficiency and mitigate risks associated with worn instruments. Overall, the scanning resolution utilized is appropriate for the number of perforations performed, enabling accurate wear measurements that would be difficult to capture using lower-resolution tools or subjective assessment methods.

**Figure 21:**
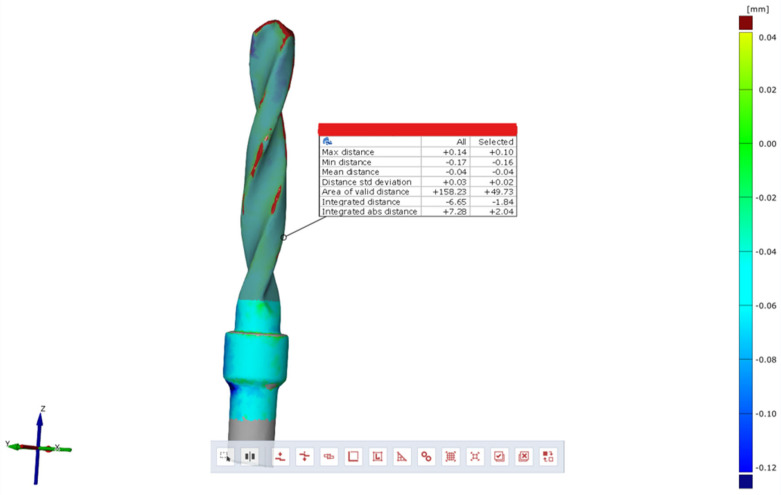
Surface comparison reveals a maximum thickness increase of +0.10 mm and a minimum thickness reduction of −0.16 mm, with an average deformation of −0.04 mm across the entire surface analyzed.

**Figure 22:**
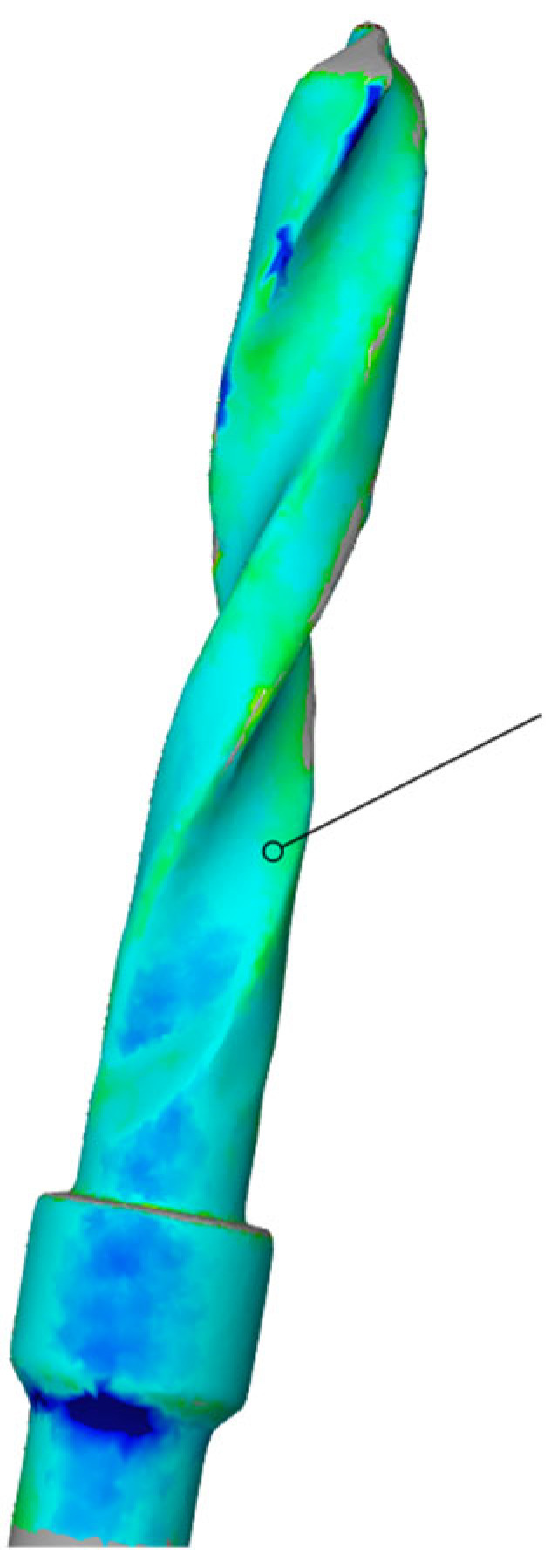
In blue, areas with increased wear are highlighted.

A comparison of the surfaces shows that the maximum increasing discrepancy (deformation that increases thickness) is +0.10 mm, while the minimum decreasing discrepancy (deformation that reduces thickness) is −0.16 mm. The average deformation observed over the entire analyzed surface is −0.04 mm (Figure 21). Areas of increased wear are highlighted in blue (Figure 22).

**Figure 23:**
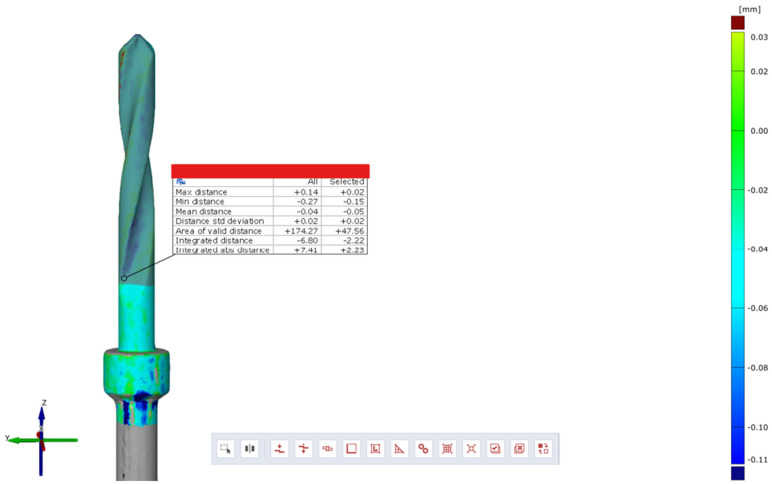
Surface comparison shows a maximum thickness increase of +0.02 mm, a minimum reduction of −0.15 mm, and an average deformation of −0.05 mm across the analyzed surface.

**Figure 24:**
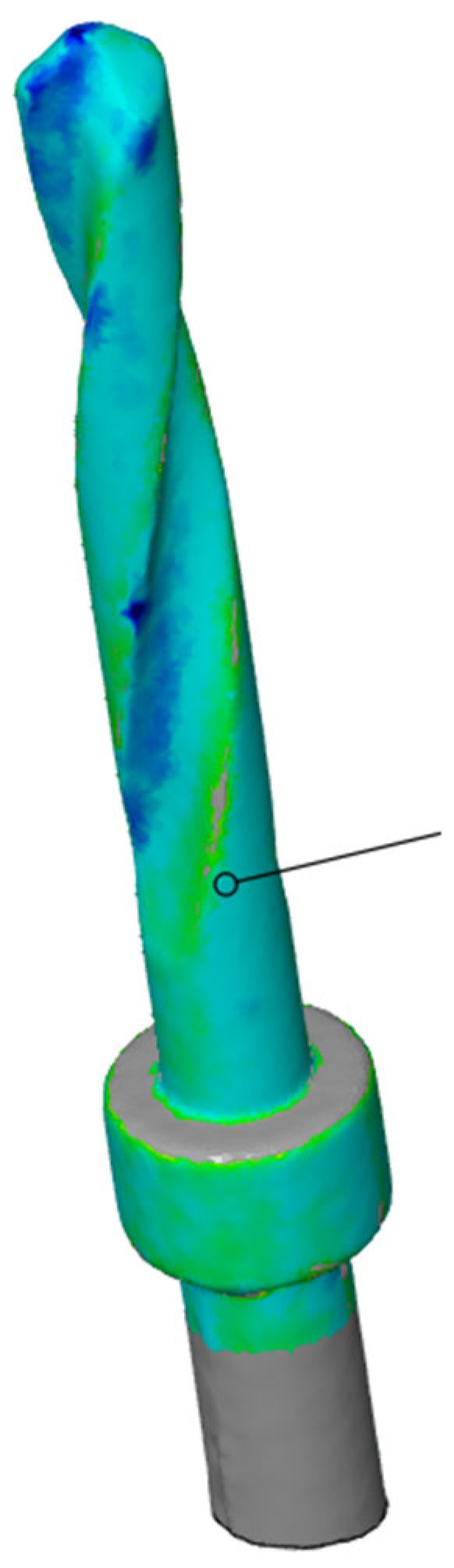
In blue, areas with increased wear are highlighted.

Comparison of pilot drill no. 1 pre- and post-use:

A comparison of the surfaces shows that the maximum increasing discrepancy (deformation that increases thickness) is +0.02 mm, while the minimum decreasing discrepancy (deformation that reduces thickness) is −0.15 mm. The average deformation observed over the entire analyzed surface is −0.05 mm (Figure 23). Areas of increased wear are highlighted in blue (Figure 24).

**Figure 25:**
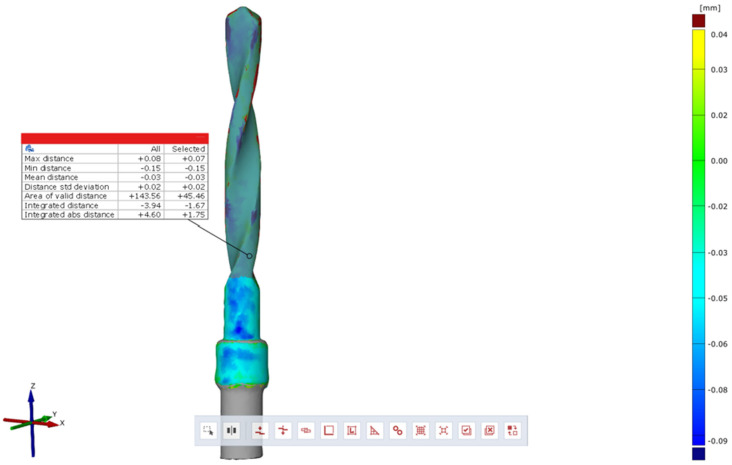
Surface comparison reveals a maximum thickness increase of +0.07 mm, a minimum reduction of −0.15 mm, and an average deformation of −0.03 mm across the analyzed surface.

**Figure 26:**
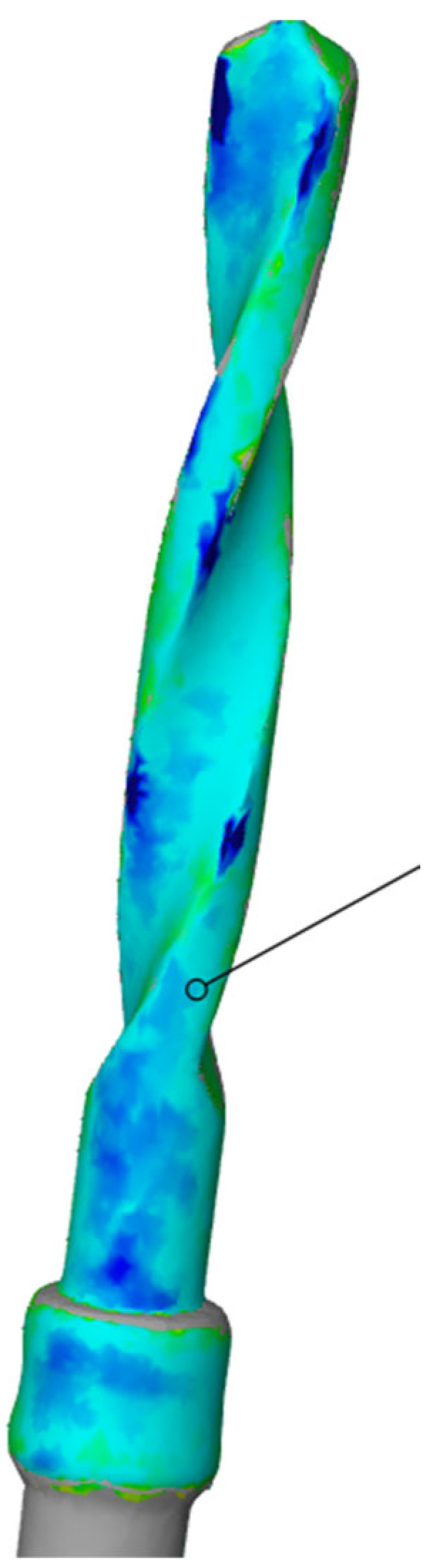
In blue, areas with increased wear are highlighted.

Comparison of pilot drill no. 2 pre- and post-use:

A comparison of the surfaces shows that the maximum increasing discrepancy (deformation that increases thickness) is + 0.07 mm, while the minimum decreasing discrepancy (deformation that reduces thickness) is − 0.15 mm. The average deformation observed over the entire analyzed surface is 0.03 mm (Figure 25). Areas of increased wear are highlighted in blue (Figure 26).

Comparison of pilot drill no. 3 pre- and post-use:

## 3. Results

Table 2 shows the results of the temperature measurements recorded after the use of the cutters with an Eventek instrument placed at a standard distance, directing infrared rays at the tip of the cutters and at 15 mm.

In addition to the temperature measurements, the time required to drill to the desired depth (15 mm) was recorded using a stopwatch. The values obtained from these measurements are shown in Table 3.

The wear of the pilot cutters was assessed by comparing three-dimensional scans taken before and after use. The scans were performed using Zeiss Inspect optical 3D software. The resulting STL files were overlaid to analyze the degree of wear in three dimensions. Surface discrepancies were quantified: the maximum increase in thickness was +0.10 mm for pilot drill no. 1, +0.02 mm for pilot drill no. 2, and +0.07 mm for pilot drill no. 3. The minimum decrease in thickness was −0.16 mm for pilot drill no. 1, and −0.15 mm for both pilot drill no. 2 and pilot drill no. 3. The average deformation across the entire analyzed surface was −0.04 mm for pilot drill no. 1, −0.05 mm for pilot drill no. 2, and −0.03 mm for pilot drill no. 3, with a consistent standard deviation across all pilot drills (Table 4).

## 4. Statistical Analysis

### 4.1. Temperature Variations

The mathematical model outlined represents the thermal processes in biomaterial drilling by integrating temperature data and wear observations. It begins with empirical heat generation, showing that pilot drill no. 1 reached the highest average temperature of 23.76 °C, compared to drills no. 2 and no. 3, which reached 23.07 °C and 23.10 °C, respectively. The heat generation can be quantified using the equation Q = μ⋅F⋅v, allowing the inference of the friction coefficient μ from the force F and drill speed v.

The temperature distribution is modeled using the heat conduction equation, incorporating specific thermal conductivities and heat capacities of the drill materials. The thermal diffusivity influences how quickly heat dissipates, with boundary conditions established from the observed maximum temperatures at a depth of 15 mm in each drill.

Heat flux at the contact surface is calculated from the temperature gradients between the drill and biomaterial, indicating that drill no. 1 has a higher heat flux due to its greater temperature. The initial conditions for the model set the starting temperature at 22.7 °C, with adjustments for heat dissipation based on the drill material.

Thermal stress is calculated using wear data, with drill no. 2 showing a significant deformation of −0.05 mm. The thermal stress equation σ_thermal_ = Eα_thermal_ΔT is applied, revealing that drill n°1, with higher thermal cycling, is likely to experience increased microcracking and fatigue stresses.

From an analysis of the data obtained from the temperature measurements, it can be concluded that pilot drill no. 1 underwent greater heating than pilot drills no. 2 and no. 3, with an average of 23.76 °C compared to 23.07 °C for pilot drill no. 2 and 23.10 °C for pilot drill no. 3. When comparing the latter two drills, no statistically significant differences were found. None of the drills, however, reached the critical temperature level (47 °C) for bone tissue necrosis.

### 4.2. Time of Use

In terms of usage time, pilot drill no. 2 took the longest time to reach the desired depth, with an average of approximately 7.93 s, compared to 7.47 s for pilot drill no. 1 and 7.754 s for pilot drill no. 3.

### 4.3. Degree of Wear

A comparison of the surfaces shows that the average deformation observed over the entire analyzed surface is −0.05 mm for pilot cutter no. 2, while lower average values were obtained for the other two pilot drills: −0.04 mm for pilot drill no. 1 and −0.03 mm for pilot drill no. 3. It can, therefore, be deduced that, on average, pilot drill no. 2 underwent greater, albeit minimal, deformation in the reduction direction when compared with the other two cutters. It is, therefore, advisable to use drills for implant site preparation in compliance with the maximum number of working cycles indicated in their respective surgical manuals to avoid compromising the cutting capacity of the instrument; it is recommended that cutters be replaced after a maximum of 15 working and sterilization cycles.

## 5. Discussion

This study’s findings align with previous research indicating that repeated use of dental drills leads to wear that impairs cutting efficiency, thermal control, and overall performance. Prior studies, such as the study of Chacon GE et al. (2006) [29] and the study of Mishra SK et al. (2014) [30], have shown that even minimal wear on drill edges increases both drilling time and the force required to reach optimal depth, which can lead to higher temperatures at the drill site. The current results, showing differential wear and associated temperature increases across the tested drills, are consistent with research on carbide- and stainless-steel-based surgical tools, where increased friction from worn edges correlates with higher operational temperatures. Unlike previous studies that focused on single drill types or limited cycles, this study presents a comparative analysis across multiple drill designs, offering broader insights into how material and design affect wear patterns and thermal management. The observed temperature rise remained below critical thresholds for bone necrosis (47 °C), supporting findings from other studies using bone analogs and cadaveric samples. However, this study’s focus on different drill types and repeated sterilization cycles advances the field by highlighting that even when temperatures are controlled, material fatigue and deformation from wear may still impair performance. This underscores the importance of following recommended drill replacement cycles, consistent with existing literature, to maintain efficient cutting and ensure patient safety. The study’s results have notable clinical implications, particularly regarding the impact of drill wear and temperature increases on implant longevity and patient outcomes. Although the observed temperature increases remained within safe limits, extended drill use could still risk localized overheating, which may interfere with osseointegration. Persistent high temperatures, as previously reported in the literature, can impede bone healing by causing microdamage to surrounding tissues, potentially compromising long-term implant stability. Additionally, the slower drilling times observed with more worn drills may necessitate greater force application, possibly impacting patient comfort and increasing surgery duration. Prolonged surgeries have been associated with higher risks of complications, such as infection and delayed healing. These findings emphasize the need for clinicians to monitor drill condition actively and adhere to the replacement cycles recommended by manufacturers, as even minor wear can impact surgical efficacy and patient recovery. By illustrating the specific effects of drill wear and temperature rise on surgical performance, the study reinforces the importance of choosing high-quality, wear-resistant materials for surgical drills and following strict maintenance protocols. These insights support the development of drills that combine high durability with minimal thermal impact, ideal for repeated clinical use. Additionally, the study suggests the potential benefit of incorporating intraoperative temperature monitoring as a preventative measure to ensure that temperatures remain within safe limits throughout the drilling process. Such monitoring, combined with regular tool replacement, could mitigate risks related to excessive wear and overheating, ultimately promoting implant longevity and improving patient outcomes. The accurate measurement of wear may also be influenced by thermal expansion, as temperature variations before and after use can slightly alter surface dimensions. Given that metal expands with heat, differences in pre- and post-use temperatures might affect the accuracy of wear measurements. To control for this, the study standardized drill cooling to ambient temperature before scanning, reducing the influence of thermal expansion on wear assessments. By maintaining consistent temperatures, wear measurements reliably reflected actual material degradation rather than artifacts due to expansion or contraction, thereby strengthening the reliability of the findings. Overall, this study contributes valuable insights into the wear and thermal properties of dental drills, aligning with existing research, while offering enhanced clinical guidance for tool maintenance and replacement. By ensuring drills are replaced before wear reaches clinically significant levels, surgical outcomes can be optimized, reducing risks to bone health and supporting robust implant longevity and patient recovery.

Temperature variations:

A detailed analysis of temperature distribution and thermal stress within the drilling zone indicates that, while average temperatures remain generally low, localized heat flux in small contact areas can produce significant temperature gradients. These gradients generate primary thermal stresses within the bulk of the drill material and secondary stresses in the interfacial layer, causing cyclical thermal strain. This thermal cycling may accelerate tool wear due to these fluctuating microthermal cycles. The concentrated heat flux at the drill tip results in sharp temperature gradients that induce distinct thermal stresses throughout the drill. Specifically, intense localized heat at the biomaterial–drill interface causes steep temperature variations, leading to first-kind thermal stresses due to the expansion and contraction of the drill material. Additionally, secondary thermal stresses arise in the interphase layer, where the thermal conductivity varies between the drill’s core and surface coatings, especially in carbide-coated drills. These thermal stresses exhibit an impulsive quality, driven by rapid and repetitive heating and cooling cycles during drilling. As the drill intermittently contacts the biomaterial, thermal energy alternately accumulates and dissipates, creating a cyclical thermal load. This process generates cyclical thermal stresses that progressively weaken the drill’s microsurface layers, forming microcracks where thermal expansion differences occur. Thermal cycling accelerates surface degradation, producing microstructural fatigue with each drilling pass. Observations through electron microscopy and spectral microanalysis confirm microcracks and other fatigue markers, supporting the thermal cycling hypothesis. These findings emphasize the need for drills with optimized thermal properties, such as improved conductivity in surface coatings, and underscore the importance of routine tool replacement to counteract the cumulative impacts of thermal cycling on durability and performance.

The analysis of temperature data indicates that pilot drill no. 1 experienced greater heating compared to pilot drills no. 2 and no. 3, with an average temperature of 23.76 °C, versus 23.07 °C for pilot drill no. 2 and 23.10 °C for pilot drill no. 3. The difference between drills no. 2 and no. 3 was not statistically significant. Importantly, none of the drills reached the critical threshold of 47 °C, which is associated with the risk of bone tissue necrosis.

Time of use:

Regarding the time required to reach the desired depth, pilot drill no. 2 demonstrated the longest average time, at 7.93 s, compared to 7.47 s for pilot drill no. 1 and 7.754 s for pilot drill no. 3. This indicates that drill no. 2 may have a marginally slower cutting performance.

Degree of wear:

The study employs electron microscopy and spectral microanalysis to gain insights into the physical wear processes affecting drilling tools. These analyses reveal several key wear mechanisms that contribute to the degradation of the tools under drilling conditions.

Adhesive wear is identified as a significant mechanism, characterized by the adherence of biomaterial particles to the drill surface, resulting in the formation of microscopic bonds that break as the drill rotates. This cyclical bonding process leads to material transfer onto the drill, increasing surface friction and thermal load, thereby accelerating wear. The model for adhesive wear emphasizes the importance of material compatibility and frictional heat in influencing wear severity.

Abrasive wear is another crucial mechanism, arising from hard particles within the biomaterial, such as mineralized deposits, which abrade the drill surface. These particles function as microcutters, creating grooves and scratches that erode the cutting edges, reducing the drill’s effectiveness over time. The abrasive wear model posits that wear intensity is determined by the hardness of these particles in relation to the drill material, with harder particles causing deeper grooves.

Thermal fatigue is caused by repeated cycles of heating and cooling during drilling, leading to the expansion and contraction of the drill surface at the drill–biomaterial interface. Over time, these thermal fluctuations can create microcracks that propagate through the surface layers, diminishing durability. The thermal fatigue model quantifies the rate of microcrack formation based on thermal cycling, temperature gradients, and the thermal expansion coefficient of the drill material, indicating that materials with high thermal resistance and low expansion coefficients are more capable of withstanding repeated thermal cycling.

Oxidative wear occurs at elevated temperatures, where oxygen interacts with the drill surface, forming oxides that initially protect the material. However, these oxide layers can become brittle and flake off under stress, resulting in progressive material loss and roughening of the surface. The oxidative wear model considers temperature and exposure time to oxygen as critical factors, suggesting that increased drilling temperatures accelerate oxidation, while intermittent cooling may reduce this effect.

Surface microcracking and delamination arise from stress accumulation at the interface between the drill coating and its substrate. Increased thermal and mechanical cycling can lead to the formation and propagation of microscopic cracks, ultimately causing coating detachment. The model addressing microcracking and delamination highlights the importance of matching the thermal expansion rates of the coating and substrate to mitigate delamination risks.

Overall, these wear models provide a framework for predicting tool degradation based on specific drilling conditions and interactions. This understanding aids in the design of more wear-resistant drills by selecting appropriate materials and coatings to counteract the identified wear mechanisms.

Surface wear analysis revealed that pilot drill no. 2 exhibited the greatest average deformation across the entire surface, with a reduction of −0.05 mm, compared to −0.04 mm for pilot drill no. 1 and −0.03 mm for pilot drill no. 3. Although minimal, these findings suggest that pilot drill no. 2 underwent slightly more deformation in the reduction direction than the other drills. These results highlight the importance of adhering to the recommended maximum number of use and sterilization cycles specified in the surgical guidelines to maintain the cutting efficiency of the drills. Based on the findings, it is recommended that pilot drills be replaced after a maximum of 15 working and sterilization cycles to avoid compromised performance.

## 6. Conclusions

This comparative study aimed to provide a comprehensive evaluation of the wear, temperature rise, and usage time of three different pilot drills used for implant site preparation, each from a different manufacturer. This study utilized pig ribs as a biological model to simulate clinical conditions, allowing for a controlled assessment of the performance of these tools. The findings offer valuable insights into the behavior of these drills, which can have direct implications for improving clinical outcomes in dental implantology, particularly in optimizing the balance of precision, efficiency, and safety during surgery. The results of this study underscore the importance of adhering to manufacturer recommendations for the replacement of dental drills after a maximum of 15 working and sterilization cycles. These recommendations are supported by clinical guidelines, which emphasize the critical role that tool sharpness, wear, and thermal control play in ensuring successful surgical outcomes. The findings highlight the need for ongoing monitoring of drill condition during clinical practice, as wear and thermal management are key factors in maintaining both the safety and efficiency of dental implant procedures. By replacing worn tools in a timely manner and being attentive to the effects of temperature rise during drilling, clinicians can significantly reduce the risk of intraoperative complications and improve patient outcomes. Furthermore, this study calls for the continued development of improved drill designs that prioritize both performance and longevity, ensuring that surgeons can operate with the utmost precision and confidence, while minimizing risks to patient health.

This comparative study provides valuable insights into the wear characteristics, thermal effects, and performance of three different pilot drills used for implant site preparation in dental implantology, each sourced from a different manufacturer. Utilizing pig ribs as a biological model allowed for a controlled assessment of these tools, simulating clinical conditions and enabling a comprehensive evaluation of their behavior. By highlighting the relationship between drill wear, temperature rise, and clinical efficiency, this study reinforces the critical importance of proper drill maintenance and timely replacement to ensure patient safety and optimal surgical outcomes.

The findings underscore the necessity of adhering to manufacturer recommendations for replacing dental drills after a maximum of 15 working and sterilization cycles. These guidelines emphasize the vital role of tool sharpness, wear, and thermal control in achieving successful surgical outcomes. Moreover, ongoing monitoring of the drill’s condition during clinical practice is essential, as wear and thermal management are key factors in maintaining both the safety and efficiency of dental implant procedures. By replacing worn tools promptly and being attentive to the effects of temperature rise during drilling, clinicians can significantly reduce the risk of intraoperative complications and enhance patient outcomes.

However, some limitations of this study must be acknowledged. One primary limitation is its in vitro nature, as it relies on pig ribs as a model for human bone. While pig bone offers a similar density and structure, it may not fully replicate the conditions encountered in human bone, particularly across different patient age groups or bone densities. Future studies could address this limitation by incorporating a wider variety of biological models, including cadaveric human bone, to provide a more comprehensive assessment of drill performance under diverse clinical conditions.

Additionally, while this study assessed drill wear and temperature changes after a controlled number of perforations, real clinical scenarios often involve varying levels of drill use and differing sterilization cycles. Extending the analysis to cover a broader range of usage cycles and sterilization frequencies could yield a deeper understanding of when and how specific types of wear begin to impact clinical safety and performance. Furthermore, investigating alternative drill materials and coatings that enhance durability and reduce heat generation—such as advanced ceramics or specialized coatings, such as diamond-like carbon (DLC) or titanium nitride (TiN)—could significantly improve both wear resistance and thermal control.

Furthermore, while this study took steps to control for thermal expansion, small temperature fluctuations might still influence wear measurements. Future research might implement even more precise temperature control mechanisms or incorporate real-time temperature monitoring technologies that allow for immediate adjustments, ensuring that temperatures remain consistently within safe limits throughout the drilling process.

In conclusion, this study not only lays important groundwork for understanding the performance of dental pilot drills but also calls for the continued development of improved drill designs that prioritize both performance and longevity. By fostering innovation and adhering to best practices in the field, clinicians can operate with the utmost precision and confidence, while minimizing risks to patient health. Ultimately, pursuing these research directions will enhance the understanding of drill performance and contribute to safer, more effective implant surgeries, thereby improving overall patient outcomes and surgical reliability.

Future research could expand on this study’s findings by exploring various strategies to improve drill durability, thermal performance, and clinical efficacy. One promising direction is the investigation of alternative materials beyond stainless steel and carbide. Materials such as advanced ceramics or metal composites could provide drills with improved wear resistance and lower thermal conductivity, reducing temperature rise during drilling and protecting bone tissue from thermal damage. Additionally, applying specialized coatings, such as diamond-like carbon (DLC) or titanium nitride (TiN), could further enhance the surface hardness and reduce edge degradation over multiple uses. These coatings might also act as thermal barriers, limiting heat transfer to the bone and lowering the risk of necrosis. Comparative studies on coated versus uncoated drills could provide valuable insights into the long-term clinical benefits of such enhancements.

Another critical area for exploration is the impact of bone density variations on drill performance. Given that bone density affects both temperature generation and drill wear rate, future studies could examine how drills perform in different bone densities, simulating various anatomical sites or patient demographics. This research could pave the way for developing drill types specifically optimized for different bone densities, allowing for more tailored approaches in implant surgeries. Additionally, understanding the biomechanical effects of different drill designs and materials on bone microstructure could help optimize drill geometry and materials, supporting quicker, more stable osseointegration.

Incorporating real-time monitoring technologies, such as temperature and torque sensors, into surgical setups could enable intraoperative adjustments to maintain safe temperatures and prevent excessive wear. These intelligent systems would provide clinicians with immediate feedback on critical thresholds, reducing the need for manual monitoring and enhancing procedural safety. Finally, extending wear analysis to cover longer usage cycles and higher sterilization frequencies could identify the specific wear types that start to affect clinical safety and performance. Such research would contribute to guidelines on maximum drill usage limits and help establish effective replacement protocols, tailored to different surgical demands.

By advancing these research directions, the field can gain a deeper understanding of the factors affecting drill performance, leading to innovations in material science and surgical technology that ultimately enhance patient outcomes and surgical precision.

## Figures and Tables

**Table 1:** Mechanisms of tool wear at the macro, meso, and micro levels and their causes.

Wear Level	Mechanism	Description	Primary Causes
Macro	Thermal Fatigue	Cyclic thermal stresses lead to gradual weakening and potential cracking of the drill at a larger scale.	High friction, repetitive thermal cycling, inadequate cooling
	Deformation	Visible bending or deformation of the drill tip and edges due to prolonged high-force use.	High drilling pressure, hard biomaterial
Meso	Surface Abrasion	Creation of grooves and scratches across the drill surface as harder particles in the biomaterial abrade it.	Contact with hard particles in biomaterial, drill speed
	Oxidative Wear	Oxidation layer formation that deteriorates under heat and repeated use.	Elevated temperatures, chemical reactions with biomaterial
Micro	Micro Cracking	Formation of microcracks due to localized thermal expansion and contraction at the tool’s surface layer.	Thermal cycling, low thermal conductivity of biomaterial
	Adhesive Wear	Bonding and subsequent detachment of biomaterial particles on the drill surface, leading to microscale wear.	Frictional heat, material adhesion, repetitive contact cycles
	Edge Blunting	Gradual loss of cutting sharpness at the drill edges, reducing precision and efficiency.	Material hardness, repeated sterilization cycles, abrasion

**Table 2:** Measurements of the temperatures reached by the cutters after use, recorded at the working length (15 mm) and at the tip.

Drill no. 1 Temperature in °C (15 mm)	Drill no. 1 Temperature in °C (Tip)	Drill no. 2 Temperature in °C (15 mm)	Drill no. 2 Temperature in °C (Tip)	Drill no. 3 Temperature in °C (15 mm)	Drill no. 3 Temperature in °C (Tip)
25.3	25.5	22.9	22.9	23.4	23.1
24.1	24.3	23.5	23	23.6	23.5
25.5	25.7	22.6	22.2	23.5	23.1
25	25.2	23.4	23.1	22.8	22.9
25	25.2	23.6	23.2	23.2	23.2
24	25.5	22.4	21.9	23.6	23.4
22.7	22.7	23.6	23.3	23.1	22.9
22.7	22.5	24	23.7	22.9	22.8
22.9	22.8	23.5	23.3	23.2	22.8
22.6	22.6	23.7	23.2	23.2	22.9
23	22.9	23.3	23.2	23.4	23.2
23.7	23	23.6	23.4	23.8	23.5
23.6	23.2	23.5	23.3	23	22.9
23.1	22.7	23	22.9	23.2	23.1
22.9	22.6	23.6	23.4	23.3	23.2

**Table 3:** Measurements of usage time of the cutters to drill to the desired working length (15 mm).

Usage Time of Drill no. 1 in Sec	Usage Time of Drill no. 2 in Sec	Usage Time of Drill no. 3 in Sec
7.72	7.84	7.59
7.4	7.61	8.82
7.79	8.28	7.88
8.23	7.67	7.36
7.35	7.41	8.3
7.35	8.85	7.28
8.26	8.01	7.99
7.85	8.15	6.91
7.24	7.41	7.23
7.74	7.85	7.99
7.24	8.18	8.26
7.24	7.54	7.98
7.59	8.31	7.83
6.51	7.82	7.74
6.61	7.97	7.15

**Table 4:** Wear assessment of pilot cutters using 3D scans.

	Drill no. 1	Drill no. 2	Drill no. 3
Max distance	0.1	0.02	0.07
Min distance	−0.16	−0.15	−0.15
Mean distance	−0.04	−0.05	−0.03
Standard deviation	0.02	0.02	0.02

## Data Availability

The original contributions presented in the study are included in the article, further inquiries can be directed to the corresponding author.
